# Comparison of efficacy of acupuncture-related therapy in the treatment of perimenopausal obesity: a network meta-analysis of randomized controlled trials

**DOI:** 10.3389/fmed.2025.1642421

**Published:** 2025-11-25

**Authors:** Lu Wang, Tianrui Lu, Luxuan Tu, Jiamei Zhuang, Yunxiang Xu, Guizhen Chen

**Affiliations:** 1 The Seventh Clinical College of Guangzhou University of Chinese Medicine, Shenzhen Bao’an Traditional Chinese Medicine Hospital, Guangzhou University of Chinese Medicine, Shenzhen, China; 2 Clinical Medical College of Acupuncture-Moxibustion and Rehabilitation, Guangzhou University of Chinese Medicine, Guangzhou, China

**Keywords:** acupuncture, obesity, perimenopause, systematic review, network meta-analysis

## Abstract

**Objective:**

This study aimed to compare the effects of acupuncture-related therapies on obese perimenopausal women through a systematic review and network meta-analysis (NMA) of randomized controlled trials (RCTs).

**Methods:**

Embase, MEDLINE, the Cochrane Library, Web of Science, China National Knowledge Infrastructure, Wanfang Data, Chongqing VIP, and SinoMed were systematically searched from the inception to December 2024. The certainty of evidence was assessed using the Grading of Recommendations, Assessment, Development, and Evaluation (GRADE) framework. Primary outcomes included body mass index (BMI) and body weight. Secondary outcomes included waist circumference (WC), body fat percentage, the Kupperman index, estradiol (E_2_), follicle-stimulating hormone (FSH), luteinizing hormone (LH), triglyceride (TG), total cholesterol (TC), high-density lipoprotein (HDL), and low-density lipoprotein (LDL).

**Results:**

Eighteen RCTs involving 1268 patients with perimenopausal obesity were included. The results of the network meta-analysis revealed that, when compared to traditional Chinese herbal medicine (HM) and Western medication (WM), multiple acupuncture therapies demonstrated superior efficacy on the overall clinical effectiveness. The analysis showed that acupoint catgut embedding demonstrated superior BMI, weight, the Kupperman index, and estradiol levels improvement. Electroacupuncture was most effective for WC reduction but was inferior to sham acupoint catgut embedding for BMI and weight. Warm needle acupuncture ranked highest for body fat percentage. The pairwise meta-analysis demonstrated that acupoint catgut embedding significantly reduced FSH and LH levels. The descriptive analysis suggested that acupoint catgut embedding and warm needle acupuncture were associated with improvements in TG and LDL Levels.

**Conclusion:**

The results showed that the acupuncture-related therapies can benefit patients by improving obesity indicators, perimenopausal symptoms, serum sex hormone levels, and blood lipid levels. Several acupuncture-related therapies may be more effective than WM or HM for perimenopausal obesity and could serve as alternative treatments, with method selection based on individual clinical needs, though confirmation through higher-quality trials is warranted.

**Systematic review registration:**

https://www.crd.york.ac.uk, identifier CRD42024516232.

## 1 Introduction

Obesity is a chronic metabolic disease resulting from multifactorial interactions, including genetic and environmental influences (1). From 1990 to 2022, the global prevalence of adult obesity increased across 188 countries (2–4). Obesity now represents a significant public health challenge, disproportionately burdening women. Excess adiposity activates inflammatory pathways, disrupts glucose and lipid metabolism, and elevates risks of endocrine disorders. Perimenopausal obesity, occurring in women aged ≥ 45 years, is characterized by significant physiological transitions during the reproductive cycle, such as declining ovarian function. These changes are frequently accompanied by psychological disturbances, including anxiety and depression (5, 6). Diminished ovarian function reduces endogenous estrogen levels while elevating follicle-stimulating hormone (FSH), impairing lipid metabolism regulation and altering the quantity, composition, and distribution of adipose tissue in perimenopausal women. Animal studies indicate that declining estrogen and rising FSH levels during reproductive aging may drive the distinct fat redistribution observed in menopausal obesity. Additionally, estrogen influences adipose tissue distribution, typically causing fat to accumulate centrally—shifting from subcutaneous to visceral layers, particularly in the hips, abdomen, and thighs (7). During perimenopause, reduced estrogen levels impair pancreatic β-cell responsiveness to glucose, compromising insulin-mediated glycemic control. This disrupts glucose oxidation and utilization, exacerbating hyperglycemia and metabolic dysregulation (8). Consequently, perimenopausal obesity-associated metabolic disturbances elevate risks of hyperlipidemia, type 2 diabetes mellitus, cardiovascular disease, breast cancer, and osteoporosis (9–12). Notably, obese menopausal or postmenopausal women face significantly higher overall mortality, with studies reporting a fourfold increase in cardiovascular-related deaths among those with a BMI > 29 kg/m^2^ (13). Effective weight and body composition management during menopause is critical for preserving women’s health. Perimenopausal interventions offer a pivotal opportunity to mitigate disease risks and enhance quality of life.

Current mainstay treatments for perimenopausal obesity include lifestyle interventions, hormone replacement therapy (HRT), and bariatric surgery. However, adherence to lifestyle interventions is frequently suboptimal, while HRT carries significant risks, including breast cancer, cardiovascular diseases (e.g., coronary heart disease and stroke), cognitive decline, venous thromboembolism, and osteoporosis (14–16). Additionally, bariatric surgery entails substantial costs, creating considerable economic burdens for patients (17–19). Consequently, complementary approaches such as acupuncture are increasingly being explored (20). As a specialized acupuncture modality, acupoint catgut embedding has demonstrated favorable outcomes for reducing adiposity and alleviating perimenopausal symptoms compared to dietary interventions and traditional Chinese medicine in previous meta-analyses (21). Nevertheless, comparative evidence remains limited regarding the relative effectiveness of distinct acupuncture methods in perimenopausal obesity or their impacts on sex hormone and blood lipid levels. Therefore, the optimal selection of acupuncture techniques for managing perimenopausal obesity in clinical practice remains unclear.

This study employed a network meta-analysis (NMA) to synthesize direct and indirect evidence regarding the efficacy of different acupuncture methods in managing perimenopausal obesity. The results provide evidence-based guidance to assist clinicians in optimizing treatment strategies for this patient population.

## 2 Materials and methods

### 2.1 Eligibility criteria

#### 2.1.1 Type of study design

This review will include only parallel-arm randomized controlled trials (RCTs). We will exclude other study designs (randomized or non-randomized). No restrictions will be imposed on the language or year of publication.

#### 2.1.2 Type of population

The patients will be required to meet clear diagnostic criteria. Obesity will be defined according to the 2016 Obesity Treatment Guidelines (22) and the “Obesity Primary Diagnosis and Treatment Guidelines (2019)” (23). Additionally, obesity will be defined as body mass index (BMI) ≥ 28 or waist circumference (WC) ≥ 80 cm. Perimenopause will be determined by World Health Organization (WHO) criteria (24), encompassing the period from the onset of ovarian functional decline to 1 year postmenopause. No restrictions will be applied to regarding nationality, race, or ethnicity.

#### 2.1.3 Type of intervention

All acupuncture methods (e.g., manual acupuncture, electroacupuncture, warm needle acupuncture, auricular acupuncture, acupoint pressure, acupoint application, acupoint electrical stimulation, acupoint catgut embedding, moxibustion, etc.) will be classified as treatment interventions. Control interventions will comprise placebo acupuncture, distinct acupuncture methods, or pharmacological therapy. Studies combining acupuncture with pharmacological or non-pharmacological interventions will be included, provided treatment and control groups receive identical concurrent therapies to minimize potential confounding factors.

#### 2.1.4 Type of outcome measure

##### 2.1.4.1 Primary outcomes

BMI and body weight will serve as primary outcomes of this study.

#### 2.1.5 Secondary outcomes

The waist circumference (WC), body fat percentage, Kupperman index, estradiol level (E_2_), follicle-stimulating hormone level (FSH), luteinizing hormone level (LH), triglyceride (TG), total cholesterol (TC), high-density lipoprotein (HDL), and low-density lipoprotein (LDL) will serve as secondary outcomes in this study.

We selected a more comprehensive and detailed report if a study was published in multiple journals.

### 2.2 Exclusion criteria

Exclusion criteria will comprise: (1) patients with obesity secondary to underlying conditions (e.g., diabetes or organic diseases); (2) studies reporting no relevant outcomes; (3) total sample sizes smaller than 30 participants; (4) combined use of multiple acupuncture methods or comparing different acupuncture points, techniques, or frequencies; (5) duplicate publications; and (6) incomplete data, even after contacting authors for clarification.

### 2.3 Literature retrieval

Electronic databases will be systematically searched for relevant clinical studies on acupuncture for perimenopausal obesity. The Chinese literature will be retrieved from four databases: China National Knowledge Infrastructure, VIP Database, WanFang, and SinoMed. Additionally, English literature will be searched through MEDLINE, Embase, Cochrane Library, and Web of Science. No restrictions will be applied to language or publication year. Reference lists of included articles and relevant reviews will be screened for additional eligible studies. The search strategies for each database are detailed in Supplementary Material 1. The search and screening process will be presented following the Preferred Reporting Items for Systematic Reviews and Meta-Analyses flow diagram.

### 2.4 Study selection and data collection

Two researchers (LT and TL) will independently perform study selection and data extraction. Any disagreements regarding inclusion or data extraction will be resolved through discussion with all authors. All search results will be imported into EndNote 20 for management. Following duplicate removal, an initial screening of titles and abstracts will be conducted. Subsequently, eligible studies will be identified through full-text assessment.

Data extracted from included studies will be imported into Microsoft Excel 2016 and will comprise study characteristics (first author, publication year, language, country, study design), population details (sample size, age, TCM pattern recognition), treatment/control interventions, quality assessments, outcome measures, and adverse events. For studies published in multiple journals, the most comprehensive version will be selected. Missing data will be addressed by contacting authors via email for clarification. Two reviewers (LT and TL) will independently verify the extracted data, with any discrepancies resolved through consensus or consultation with a third reviewer.

### 2.5 Risk of bias assessment

The Cochrane Risk of Bias Tool (25) will be independently applied by two reviewers (LT and TL) to evaluate study quality. The risk of bias will be assessed based on the following domains: randomization method, allocation concealment, blinding of patients, investigators and outcome assessors, completeness of outcome data, selective reporting of results, and other potential biases. Each domain will be categorized as low, high, or unclear risk. Final evaluations will be cross-verified by both reviewers, with any unresolved discrepancies adjudicated by a third reviewer.

### 2.6 Data analysis and synthesis

#### 2.6.1 Conventional pairwise meta-analysis

Conventional pairwise meta-analyses will be conducted for direct comparisons using Review Manager 5.4 when studies exhibit clinical homogeneity in population, interventions, and outcomes. For continuous outcomes, weighted mean difference (MD) with 95% confidence intervals (CIs) will served as the effect measure to pool numerical data. Statistical heterogeneity will be assessed using the I^2^ statistic and corresponding *P*-values. A fixed-effects model will be employed if *P* ≥ 0.10 and *I*^2^ ≤ 50%, whereas a random-effects model will be applied if *P* < 0.10 and *I*^2^ > 50%.

#### 2.6.2 Network meta-analysis

Before NMA, the transitivity assumption and clinical similarity will be secured through an independent verification process where two reviewers will systematically assess clinical and methodological homogeneity across studies, evaluating key domains including study design, population characteristics, intervention protocols, and outcome measurements.

Network meta-analysis (NMA) will be conducted under a frequentist framework using the network package in Stata/MP 17.0. The network package is a suite of programs for importing data for network meta-analysis, running a contrast-based network meta-analysis using mvmeta or metareg, assessing inconsistency, and graphing the data and results. To account for the correlations arising from multi-arm trials, the model inherently employs a multivariate meta-analysis approach that correctly handles the covariance between effect estimates sharing a common control group.

First, we will employ the network map command to generate a network evidence diagram after defining the network structure with network setup, which will illustrate the quantitative relationships among individual interventions. Network diagrams will depict intervention relationships, with node sizes proportional to sample populations and line thickness reflecting trial counts. Second, we will fit both an inconsistency model and a consistency model using the network meta i and network meta c commands, respectively. Third, global inconsistency will be evaluated with the network forest command, while node-splitting analysis via network sidesplit and loop-specific inconsistency assessed with ifplot will be applied to examine disagreements between direct and indirect evidence within closed loops of three treatments. Consistency in study design will be verified through both local and global methods to ensure the robustness of the findings. Fourth, for result visualization, the intervalplot command will be used to generate a forest plot displaying effect estimates and 95% CIs, and the netleague command will be utilized to present NMA results in a league table and an inverted triangle diagram based on pairwise comparisons. Fifth, treatment rank probabilities will be calculated using the network rank and sucra prob commands. Interventions will be ranked via Surface Under the Cumulative Ranking Curve (SUCRA) values, with cumulative probability curves illustrating optimal intervention hierarchies (higher SUCRA indicating superior efficacy). Sixth, the comparison-adjusted funnel plot will be created for analyses involving ≥ 10 studies with netfunnel command to evaluate potential publication bias and small-study effects.

### 2.7 Quality of evidence

The quality of evidence for direct, indirect, and network meta-analysis (NMA) effect estimates will be assessed using the Grading of Recommendations, Assessment, Development, and Evaluation (GRADE) framework (26). Direct evidence will be evaluated for risk of bias, indirectness, inconsistency, imprecision, and publication bias, with ratings categorized as high, moderate, low, or very low. The lowest ratings of the two direct comparisons forming the most dominant first-order loop and intransitivity will be used to rate the indirect estimate. The NMA evidence rating will be determined by the highest quality level between direct and indirect evidence. For our primary outcomes, conclusions from the NMAs will be drawn by considering the effect estimates, quality of evidence (according to the GRADE approach), and treatment rankings (i.e., SUCRA) based on the partially contextualized GRADE framework (27).

## 3 Results

### 3.1 Literature search and results

On February 6, 2024, eight English and Chinese databases (MEDLINE, Embase, Cochrane Library, Web of Science, China National Knowledge Infrastructure, VIP Database, WanFang, and SinoMed) were systematically searched. The literature search was updated and re-executed in December 2024 to incorporate recent evidence. No restrictions were imposed on language or publication year. Reference lists of included articles, including relevant reviews, were screened for additional eligible studies. Search strategies and results for each database are outlined in Supplementary Material 1.

**FIGURE 1:**
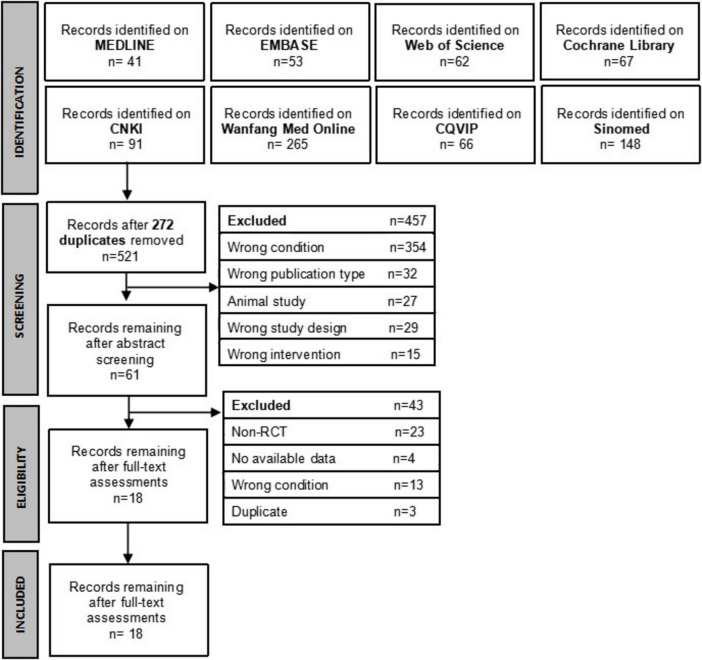
Literature screening process and results.

In total, 793 related articles were searched through electronic databases. After deleting 272 duplicates, the title and abstract of 521 records were screened, and 460 records were excluded. The full text of the remaining 61 records was reviewed, among which 43 were excluded. Therefore, 18 RCTs (5, 4, 28–44), including 1268 perimenopausal obese patients, were included in this review. The flowchart of literature screening and results are shown in Figure 1.

### 3.2 Study characteristics

Of the 18 included studies, two were written in English, and the others were published in Chinese. Eleven studies reported pattern recognition in patients. Most of them were related to spleen and stomach dampness, and there were syndromes related to spleen and kidney deficiency, liver stagnation, and qi stagnation. In total, 12 studies used acupoint catgut embedding (ACE). Electroacupuncture (EA) was used in 3 studies, warm needle acupuncture (WNA) was used in 2 studies, moxibustion (Moxa) was used in 1 study, and sham acupoint catgut embedding (SA) was used in 1 study. Non-medical interventions, including lifestyle intervention and dietary control, were used in 11 studies. Conventional estrogen therapy with nilestriol was used in four studies. Traditional Chinese herbal medicine (HM) was used in 3 studies. Six studies reported that institutional review board approval was obtained before the study. The characteristics of the included studies are shown in Supplementary Material 2.

### 3.3 Risk of bias within individual studies

**FIGURE 2:**
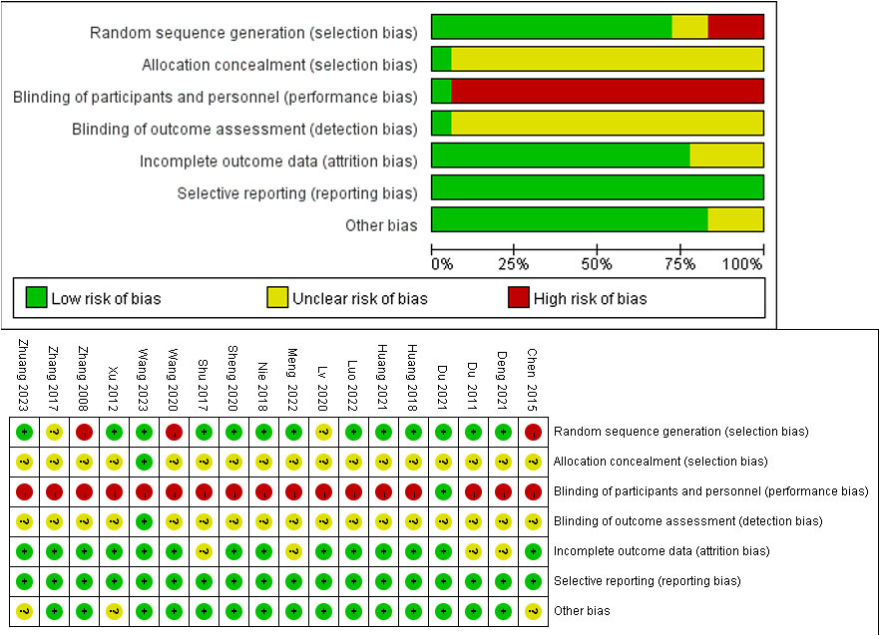
Risk of bias summary for all included studies. Low, unclear, and high risk of bias, respectively, are represented with the following symbols: “+,” “?,” and “- .”

Thirteen studies used appropriate sequence generation methods, such as random number tables, and were assessed as low risk in the domain of random sequence generation. Randomization by visit order was used in 3 studies and was assessed as high risk. Two studies did not report the precise randomization methods. One study reported distribution concealment using opaque sealed envelopes, and 17 studies did not mention the concealment method. Regarding the patient blinding process, only one study reported blinding patients, and due to limitations in intervention measures, other studies had a high risk in this regard. Only one study mentioned blinding data collectors and statistical analysts. The risk of blinding outcome assessors was unknown in other studies. All studies had a low risk regarding reporting bias. All studies reporting baseline statistical homogeneity between the two groups were assessed as having a low risk. The details of the study assessment are listed in Supplementary Material 2. The specific risk of bias is shown in Figure 2.

### 3.4 Results of pairwise meta-analysis and NMA

#### 3.4.1 Body mass index

**FIGURE 3:**
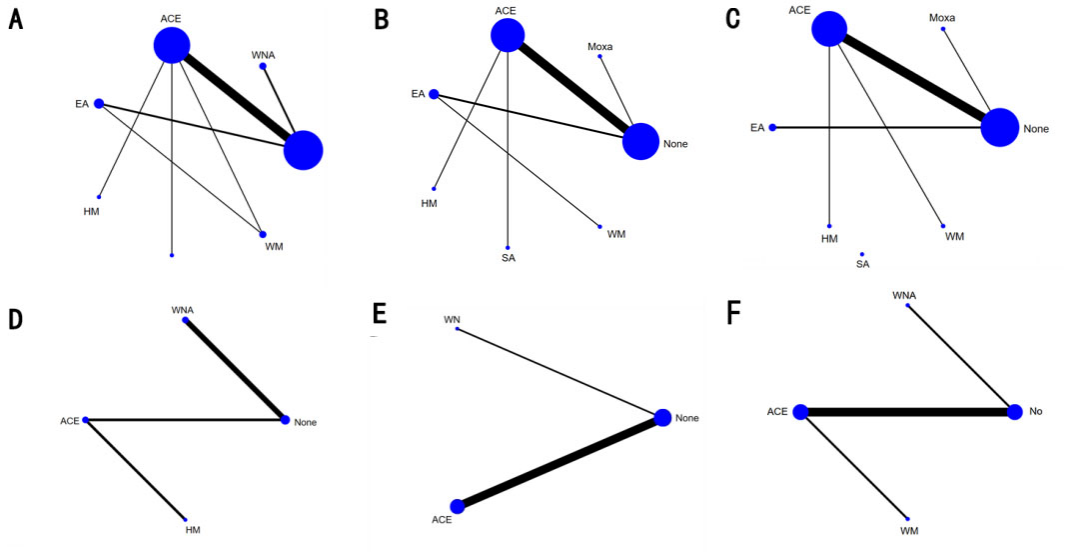
Network diagram of different indexe streated by different types of intervention. The figure shows that each intervention is indicated with a single blue dot. The size of the dot represents the cumulative total sample size of the intervention. The line segment between the dots represents the studies that have a direct comparison between the two interventions. **(A)** Body mass index, **(B)** weight, **(C)** waist circumference, **(D)** body fat percentage, **(E)** Kupperman, and **(F)** E2. ACE, acupoint catgut embedding; EA, electroacupuncture; WNA, warm needle acupuncture; SA, sham acupoint catgut embedding; HM, traditional Chinese herbal medicine; WM, Western medicine; None, no treatment.

Seventeen studies with 1,202 patients and seven intervention measures were included in the NMA of BMI, forming a closed loop (Figure 3A). The results of the inconsistent model were similar to those of the consistent model (*P* = 0.6174 > 0.05). The node-splitting method showed no significant inconsistency (*P* > 0.05), and the loop inconsistency showed no significant inconsistency (*P* = 0.81 > 0.05) (Supplementary Material 3). These findings indicated that the stability and consistency of the results were good; thus, a consistent fixed-effects model was used. In a conventional pairwise meta-analysis, acupoint catgut embedding and warm needle acupuncture were associated with a statistically significant reduction in BMI. In contrast, there was no significant difference when using electroacupuncture. In NMA, the results of acupoint catgut embedding, warm needle acupuncture, and electroacupuncture (no significant difference) were consistent with the results of the pairwise meta-analysis. Moreover, in NMA, a significant decrease was observed in BMI in the following comparisons: (a) acupoint catgut embedding therapy compared with traditional Chinese herbal medicine treatment; (b) acupoint catgut embedding therapy and warm needle acupuncture compared with western medicine treatment; (c) acupoint catgut embedding, and warm needle acupuncture compared with electroacupuncture; and (d) acupoint catgut embedding compared with sham acupoint catgut embedding (Table 1).

**TABLE 1:** League table for pairwise meta-analysis (right upper part) and NMA (left lower part) effect estimates regarding body mass index.

ACE	–	**-1.09 (-2.04, -0.14)**	–	**-2.24 (-2.64, -1.84)**	**-2.64 (-4.98, -0.30)**	**-3.71 (-6.59, -0.83)**
-0.34 (-1.08, 0.41)	WNA	–	–	**-1.91 (-2.53, -1.28)**	–	–
**-1.09 (-2.04, -0.14)**	**-0.75 (-1.96, 0.45)**	SA	–	–	–	-
**-1.48 (-2.42, -0.54)**	**-1.14 (-2.21, -0.08)**	-0.39 (-1.73, 0.95)	EA	-0.76 (-1.62, 0.09)	–	**-1.42 (-2.37, -0.47)**
**-2.24 (-2.64, -1.84)**	**-1.91 (-2.53, -1.28)**	**-1.15 (-2.18, -0.12)**	-0.76 (-1.62, 0.09)	None	–	–
**-2.64 (-4.98, -0.30)**	-2.30 (-4.76, 0.15)	-1.55 (-4.08, 0.98)	-1.16 (-3.68, 1.36)	-0.40 (-2.77, 1.98)	HM	–
**-3.71 (-6.59, -0.83)**	**-3.37 (-6.35, -0.40)**	**-2.62 (-5.65, 0.41)**	**-2.23 (-5.26, 0.80)**	0.81 (-2.37, 3.99)	-1.07 (-4.78, 2.64)	WM

The number of the ladder table is the relative effect value and 95% CI. The comparison must be read from left to right. A mean difference less than zero indicates that treatment on the left is favored in both pairwise and network meta-analyses. Bold value means a significant difference between the groups. ACE, acupoint catgut embedding; EA, electroacupuncture; WNA, warm needle acupuncture; SA, sham acupoint catgut embedding; HM, traditional Chinese herbal medicine; WM, Western medicine; None, no treatment.

**FIGURE 4:**
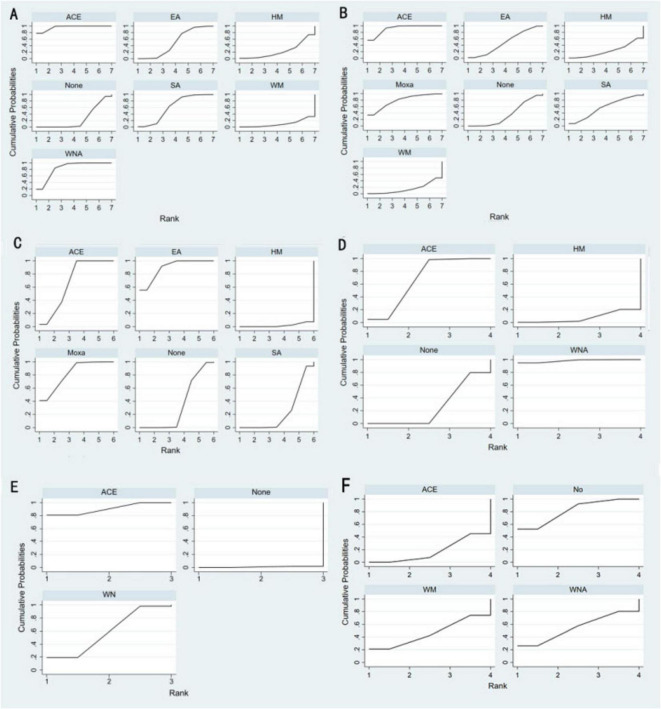
Efficacy ranking and cumulative probability graph treated by different types of intervention. **(A)** Body mass index, **(B)** weight, **(C)** waist circumference, **(D)** body fat percentage, **(E)** Kupperman, and **(F)** E2. ACE, acupoint catgut embedding; EA, electroacupuncture; WNA, warm needle acupuncture; SA, sham acupoint catgut embedding; HM, traditional Chinese herbal medicine; WM, Western medicine; None, no treatment.

According to SUCRA (Figure 4), acupoint catgut embedding was the best intervention to reduce BMI (96.3%), followed by warm needle acupuncture (83.6%), sham acupoint catgut embedding (61.0%), and electroacupuncture (50.0%).

#### 3.4.2 Body weight

NMA included 15 studies with 1,040 patients and included seven intervention measures in the analysis, which failed to form a closed loop (Figure 3B). No significant heterogeneity or inconsistency was found within the reticular body of evidence; therefore, a consistent fixed-effects model was used. According to pairwise meta-analyses, acupoint catgut embedding and moxibustion were associated with significant reductions in body weight. However, in NMA, only acupoint catgut embedding showed significant reductions in body weight. In NMA, acupoint catgut embedding significantly reduced body weight compared with traditional Chinese herbal medicine treatment and conventional Western medicine (Table 2).

**TABLE 2:** League table for pairwise meta-analysis (right upper part) and NMA (left lower part) effect estimates regarding body weight.

ACE		-2.66 (-5.54, 0.22)		**-4.11 (-5.83, -2.39)**	**-5.64 (-9.55, -1.73)**	
-0.90 (-5.51, 3.72)	Moxa			**-3.31 (-6.35, -0.27)**		
**-**2.66 (**-**6.87, 1.55)	**-**1.76 (**-**8.01, 4.48)	SA				
-3.46 (-7.12, 0.19)	-2.57 (-7.99, 2.86)	-0.80 (-6.38, 4.77)	EA	-0.70 (-3.10, 1.71)		**-2.80 (-5.39, -0.21)**
**-4.21 (-5.82, -2.59)**	-3.31 (-7.63, 1.01)	-1.55 (-6.05, 2.96)	-0.74 (-4.02, 2.53)	None		
**-5.64 (-10.62, -0.66)**	-4.74 (-11.53, 2.04)	-2.98 (-9.50, 3.54)	-2.18 (-8.35, 4.00)	-1.43 (-6.67, 3.80)	HM	
**-6.26 (-11.70, -0.83)**	-5.37 (-12.12, 1.38)	-3.60 (-10.48, 3.27)	-2.80 (-6.82, 1.22)	-2.06 (-7.24, 3.13)	-0.62 (-7.99, 6.75)	WM

The number of the ladder table is the relative effect value and 95% CI. The comparison must be read from left to right. A mean difference less than zero indicates that treatment on the left is favored in both pairwise and network meta-analyses. Bold value means a significant difference between the groups. ACE, acupoint catgut embedding; EA, electroacupuncture; SA, sham acupoint catgut embedding; Moxa, moxibustion; HM, traditional Chinese herbal medicine; WM, Western medicine; None, no treatment.

SUCRA (Figure 4) indicated that acupoint catgut embedding is the best treatment method for weight loss (91.4%), followed by moxibustion (78.0%), sham acupoint catgut embedding (57.1%), and electroacupuncture (49.2%). The comparison-adjusted funnel plot was not completely symmetrical, and there might be a risk of publication bias.

#### 3.4.3 Waist circumference

NMA included 14 studies with 980 patients and six interventions. They failed to form a closed loop (Figure 3C). No significant heterogeneity or inconsistency was found within the reticular body of evidence; therefore, a consistent fixed-effects model was employed. In pairwise meta-analysis and NMA, electroacupuncture, moxibustion, and acupoint catgut embedding were associated with significant reductions in WC. Moreover, in NMA, compared with traditional Chinese herbal medicine and sham acupoint catgut embedding, WC was significantly reduced after treatment with electroacupuncture, moxibustion, and acupoint catgut embedding (Table 3).

**TABLE 3:** League table for pairwise meta-analysis (right upper part) and NMA (left lower part) effect estimates regarding WC.

EA			**-4.82 (-6.96, -2.68)**		
**-**0.41 (-4.62, 3.80)	Moxa		**-4.41 (-8.04, -0.78)**		
-1.39 (-3.60, 0.82)	-0.98 (-4.65, 2.69)	ACE	**-3.43 (-4.00, -2.87)**	**-4.12 (-6.15, -2.09)**	**-7.64 (-11.78, -3.50)**
**-4.82 (-6.96, -2.68)**	**-4.41 (-8.04, -0.78)**	**-3.43 (-4.00, -2.87)**	None		
**-5.51 (-8.51, -2.51)**	**-5.10 (-9.29, -0.90)**	**-4.12 (-6.15, -2.09)**	-0.69 (-2.79, 1.42)	SA	
**-9.03 (-13.72, -4.33)**	**-8.62 (-14.15, -3.08)**	**-7.64 (-11.78, -3.50)**	**-4.21 (-8.38, -0.03)**	-3.52 (-8.13, 1.09)	HM

The number of the ladder table is the relative effect value and 95% CI. The comparison must be read from left to right. A mean difference less than zero indicates that treatment on the left is favored in both pairwise and network meta-analyses. Bold value means a significant difference between the groups. ACE, acupoint catgut embedding; EA, electroacupuncture; SA, sham acupoint catgut embedding; Moxa, moxibustion; HM, traditional Chinese herbal medicine; None, no treatment.

SUCRA (Figure 4) showed that electroacupuncture had the best effect (89.5%), followed by moxibustion (82.1%), acupoint catgut embedding (68.1%), and sham acupoint catgut embedding (24.1%). The symmetry of the comparison-adjusted funnel plot showed no risk of publication bias.

#### 3.4.4 Body fat percentage

NMA included four studies with 296 patients and four intervention measures, which failed to form a closed loop (Figure 3D). No significant heterogeneity or inconsistency was found within the reticular body of evidence; therefore, a consistent fixed-effects model was employed. According to pairwise meta-analysis and NMA analysis, acupoint catgut embedding and warm needle acupuncture significantly reduced body fat percentage compared with no treatment, and acupoint catgut embedding significantly reduced body fat percentage compared with traditional Chinese herbal medicine. In NMA, warm needle acupuncture reduced body fat percentage compared to traditional Chinese herbal medicine (Table 4).

**TABLE 4:** League table for pairwise meta-analysis (right upper part) and NMA (left lower part) effect estimates regarding body fat percentage.

WNA		**-3.71 (-4.94, -2.48)**	
-1.51 (-3.33, 0.31)	ACE	**-2.20 (-3.54, -0.86)**	**-3.84 (-7.41, -0.27)**
**-3.71 (-4.94, -2.48)**	**-2.20 (-3.54, -0.86)**	None	
**-5.35 (-9.36, -1.34)**	**-3.84 (-7.41, -0.27)**	-1.64 (-5.46, 2.18)	HM

The number of the ladder table is the relative effect value and 95% CI. The comparison must be read from left to right. A mean difference less than zero indicates that treatment on the left is favored in both pairwise and network meta-analyses. Bold value means a significant difference between the groups. ACE, acupoint catgut embedding; WNA, warm needle acupuncture; HM, traditional Chinese herbal medicine; None, no treatment.

According to SUCRA (Figure 4), warm needle acupuncture was the best intervention for reducing body fat percentage (98.2%), followed by acupoint catgut embedding (67.7%). However, the number of studies was small.

#### 3.4.5 Kupperman

NMA included six studies with 478 patients. The following three intervention measures were included in the analysis: acupoint catgut embedding, warm needle acupuncture, and no treatment (Figure 3E). No significant heterogeneity or inconsistency was found within the reticular body of evidence; therefore, a consistent fixed-effects model was employed. According to pairwise meta-analysis and NMA, acupoint catgut embedding and warm needle acupuncture significantly reduced Kupperman compared to no treatment (Table 5). According to SUCRA (Figure 4), acupoint catgut embedding was the best intervention for reducing BMI (90.4%), followed by warm needle acupuncture (58.6%). However, the number of studies is small.

**TABLE 5:** League table for pairwise meta-analysis (right upper part) and NMA (left lower part) effect estimates regarding Kupperman.

ACE		**-5.63 (-7.81, -3.44)**
-1.89 (-6.23, 2.46)	WNA	**-3.79 (-5.14, -2.44)**
**-5.68 (-8.15, -3.20)**	**-3.79 (-7.36, -0.22)**	None

The number of the ladder table is the relative effect value and 95% CI. The comparison must be read from left to right. A mean difference less than zero indicates that treatment on the left is favored in both pairwise and network meta-analyses. Bold value means a significant difference between the groups. ACE, acupoint catgut embedding; WNA, warm needle acupuncture, None, no treatment.

#### 3.4.6 Other outcomes of interest

NMA included six studies with 544 patients and four intervention measures, which failed to form a closed loop (Figure 3F) regarding E2. The following four intervention measures were included in the analysis: acupoint catgut embedding, warm needle acupuncture, western medicine, and no treatment. No significant heterogeneity or inconsistency was found within the reticular body of evidence; therefore, a consistent fixed-effects model was employed. According to pairwise meta-analysis and NMA, acupoint catgut embedding increased E2 compared to no treatment (Table 6). The results of descriptive analysis showed that acupoint catgut embedding was superior to Western medicine (*P* < 0.05), as shown in Supplementary Material 3. According to SUCRA (Figure 4), acupoint catgut embedding was the best intervention for reducing BMI (82.6%), followed by warm needle acupuncture (53.8%). However, the number of studies is small.

**TABLE 6:** League table for pairwise meta-analysis (right upper part) and NMA (left lower part) effect estimates regarding E2.

ACE	**11.27(8.52, 14.02)**		**27.9(9.6, 46.19)**
11.43 (-29.77, 52.64)	WM	**-3.79 (-5.14, -2.44)**	
17.15 (-28.99, 63.30)	5.72 (-56.10, 67.53)	WNA	10.71(8.83, 12.59)
**27.86 (7.14, 48.59)**	16.43 (-29.63, 62.48)	10.71 (-30.52, 51.94)	None

The number of the ladder table is the relative effect value and 95% CI. The comparison must be read from left to right. A mean difference less than zero indicates that treatment on the left is favored in both pairwise and network meta-analyses. Bold value means a significant difference between the groups. ACE, acupoint catgut embedding; WNA, warm needle acupuncture; WM, Western medicine; None, no treatment.

Only pairwise meta-analysis and descriptive analysis were possible for the following secondary outcomes because transitivity and consistency could not be examined in the network: FSH, LH, Triglyceride (TG), low-density lipoprotein (LDL), high-density lipoprotein (HDL) and total cholesterol (TC). In the direct comparison, acupoint catgut embedding significantly reduced LSH and LH after treatment compared to no treatment. The descriptive analysis results indicated that acupoint catgut embedding and warm needle acupuncture significantly reduced TG and LDL compared to no treatment. In contrast, there were no significant differences in the efficacy of TC and HDL, as shown in Supplementary Material 3.

### 3.5 Publication bias

**FIGURE 5:**
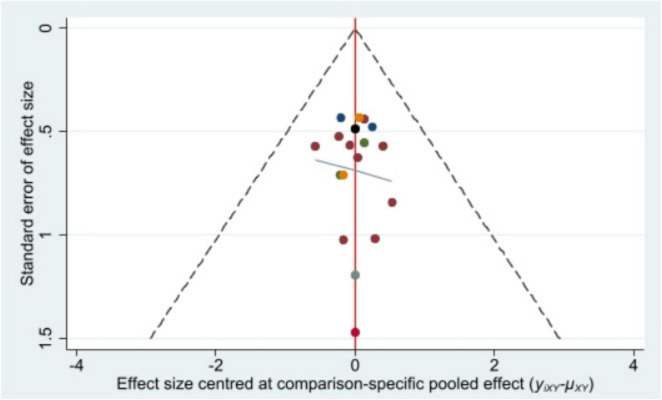
Comparison-adjusted funnel plot of the effect size.

Stata/MP 17.0 was used to plot the comparison-adjusted funnel plot for improvement of BMI, the primary outcome indicator. The comparison-adjusted funnel plot (Figure 5) showed a symmetrical distribution and no publication bias.

### 3.6 Quality of evidence

The GRADE method indicated that direct, indirect, and mixed evidence quality was “low” to “moderate” (Supplementary Material 4). The main reasons for downgrading were the high risk of bias and imprecision due to wide Cl or small sample sizes. According to the GRADE partially contextualized framework, when all interventions are considered, acupoint catgut embedding might have a large beneficial effect on BMI and body weight, warm needle acupuncture might have a moderate beneficial effect on BMI, and moxibustion might have a moderate beneficial effect on body weight. Additionally, sham acupoint catgut embedding might have a small beneficial effect on BMI and body weight (Tables 7, 8).

**TABLE 7:** GRADE approach using a partially contextualized framework regarding BMI.

Classification of intervention	Intervention	Effect estimates compared with no treatment [MD (95% CI)]	SUCRA (%)	Quality of evidence
Large beneficial effect	Acupoint catgut embedding	-2.24 (-2.64, -1.84)	96.3	Moderate
Moderate beneficial effect	Warm needle acupuncture	-1.91 (-2.53, -1.28)	83.6	Moderate
Small beneficial effect	Sham acupoint catgut embedding	-1.15 (-2.18, -0.12)	61.0	Moderate
Trivial or no effect	Electroacupuncture	-0.76 (-1.62, 0.09)	50.0	Low

**TABLE 8:** GRADE approach using a partially contextualized framework regarding body weight.

Classification of intervention	Intervention	Effect estimates compared with no treatment [MD (95% CI)]	SUCRA (%)	Quality of evidence
Large beneficial effect	Acupoint catgut embedding	-4.21 (-5.82,-2.59)	91.4	Moderate
Moderate beneficial effect	Moxibustion	-3.31 (-7.63, 1.01)	78.0	Low
Small beneficial effect	Sham acupoint catgut embedding	-1.55 (-6.05, 2.96)	57.1	Low
Trivial or no effect	Electroacupuncture	-0.74 (-4.02, 2.53)	49.2	Low

In summary, this study analyzed 18 randomized controlled trials (RCTs) involving 1,268 women with perimenopausal obesity, examining seven treatment options, including electroacupuncture, warm needle acupuncture, acupoint catgut embedding, moxibustion, traditional Chinese medicine, and Western medicine. We calculated the effect estimates between individual treatment options or compared them with conventional treatments through an NMA for the outcomes of interest and calculated the SUCRA-based treatment ranking. However, most of the evidence evaluated according to the GRADE approach was “moderate” to “low” in quality, and there was no high-quality evidence. As a result, ACE was the most effective treatment for BMI and weight. ACE outperformed WNA, EA, HM, and WM. Regarding the waist circumference after treatment, a secondary outcome is that EA was most effective, surpassing moxibustion, ACE, and HM. WNA was more effective in reducing body fat percentage, than ACE and HM. ACE was also most effective in improving Kupperman Index scores, exceeding WM. Other secondary outcomes: (1) ACE was most effective at improving E2 levels, better than WNA and WM. (2) Most studies did not report Other secondary outcomes such as the LH, LSH, TG, TC, LDL, and HDL; therefore, only pairwise meta-analysis was possible. Acupoint embedding significantly reduced LH and LSH levels compared with conventional treatments. Acupoint embedding and warm needle acupuncture notably reduced TG and LDL, though effects on TC and HDL were insignificant. These results suggest that while there are variances in efficacy across different indicators, ACE is highly effective in treating perimenopausal obesity, with no serious adverse reactions, making it suitable for clinical use.

## 4 Discussion

This study focuses on several critical health parameters in perimenopausal women, including BMI, body weight, waist circumference, body fat percentage, perimenopausal symptoms, estrogen-related indicators, and blood lipid levels. Fluctuations in these parameters significantly impact women’s health during the menopausal transition. Previous research (46) has demonstrated that even a modest weight gain of 2.5–5 kg in middle-aged women correlates with significantly elevated risks of chronic diseases and premature mortality, including diabetes, cardiovascular diseases, obesity-related cancers, and all-cause mortality. Clinical evidence (12) indicates that perimenopausal obese women achieving a body fat reduction exceeding 25% demonstrate healthier adipose distribution patterns and reduced risk of type 2 diabetes. The association between weight gain and adverse cardiac structural and functional changes is particularly notable, with the degree of BMI elevation and duration of exposure contributing to progressive cardiac dysfunction (46, 47). Notably, epidemiological studies (48) report that middle-aged women experiencing annual weight gain exceeding 0.75 kg have a 35% increased breast cancer risk, with abdominal obesity exhibiting a stronger association with breast cancer risk than overall obesity. Park et al. (49) quantified this relationship, demonstrating that each 10 cm increase in waist circumference corresponded to a 1.13-fold increase in postmenopausal breast cancer risk, suggesting waist circumference may better reflect visceral fat’s carcinogenic potential than BMI. These findings highlight the perimenopausal period as a critical window for weight management and visceral fat reduction to mitigate the risks of various diseases. The perimenopausal period is characterized by marked estrogen decline, accelerating bone loss and increasing the risk of postmenopausal osteoporosis. Research suggests maintaining BMI within 22–25 kg/m^2^ mitigates the rate of bone density reduction (50). Additionally, moderate weight loss in perimenopausal obese women alleviates joint load, thereby potentially reducing exercise-related injury risks (51).

Acupuncture is a globally recognized therapeutic intervention demonstrating clinical efficacy in improving perimenopausal obesity. Research indicates that acupuncture may modulate hypothalamic activity, establishing a neuroregulatory circuit for appetite control that could improve perimenopausal obesity (52). Evidence further suggests that acupuncture regulates fatty acid metabolism while elevating serum levels of high-density lipoprotein and carnitine (53). Mechanistically, research hypothesize that acupuncture may act through AMPK-mediated signaling pathways, though this remains speculative and requires direct validation in future studies (54). Additionally, acupuncture has been shown to alleviates perimenopausal psychological symptoms including anxiety, panic, and insomnia (55, 56), while potentially reducing risks of chronic metabolic comorbidities, including hyperlipidemia and type 2 diabetes, in this population (57, 58).

ACE involves implanting absorbable surgical sutures at acupoints to provide more sustained stimulation compared to conventional acupuncture. Its prolonged therapeutic effect may be attributed to proteolytic enzyme activity and macrophage-mediated responses to the absorbable material, which collectively enhance and extend acupoint stimulation (59). ACE has shown potential in managing perimenopausal obesity through a proposed mechanism involving enhanced adipose tissue energy metabolism. This may be mediated through increased cyclic adenosine monophosphate levels and upregulation of uncoupling protein 1 expression in brown adipose tissue (60). Furthermore, ACE appears to modulate the hypothalamic-pituitary-gonadal axis, elevate serum estradiol and progesterone levels, and consequently attenuate obesity progression (41). Experimental evidence from ovariectomized rat models indicates ACE’s superior effect to estrogen therapy in improving lipid metabolism disorders induced by estrogen deficiency (61). Our findings (Tables 1, 2, 5, 6) suggest ACE significantly improves BMI, body weight, perimenopausal symptoms, and estradiol levels, potentially establishing it as a promising therapeutic strategy for perimenopausal obesity based on current evidence.

EA constitutes an enhanced form of traditional acupuncture that employs low-frequency pulsed currents at acupoints, synergistically integrating mechanical and electrical stimulation to enhance treatment outcomes. Regarding waist circumference (WC) reduction, EA demonstrated superior efficacy (Table 3). However, its lack of significant effect on BMI suggests its particular suitability for managing abdominal obesity in perimenopausal women with normal body mass index. Moxibustion represents a clinically relevant intervention for perimenopausal obesity, encompassing Traditional Chinese Medicine (TCM) modalities, including direct moxibustion, ginger-separated moxibustion, garlic-separated moxibustion, and warm needle acupuncture (62). Moxibustion may contribute to weight reduction in obese perimenopausal women by promoting localized hemodynamics and enhancing energy metabolism (63). This effect could be mediated through the browning of white adipose tissue (64). Additionally, moxibustion appears to support ovarian function by inhibiting apoptosis in aging ovarian tissue and strengthening antioxidant capacity, leading to normalized hormone secretion (65). WNA integrates moxibustion and acupuncture techniques by combining mechanical, thermal, and acupoint stimulation. This modality is widely adopted in Asian clinical practice (66). Our analysis identified WNA as particularly effective for body fat percentage reduction (Table 4).

Although menopausal hormone therapy (MHT) and behavioral interventions can improve BMI and perimenopausal symptoms, lifestyle modifications cannot achieve adequate weight control in all patients. Moreover, MHT is associated with elevated risks of breast cancer, coronary heart disease, stroke, cognitive impairment, venous thromboembolism, and osteoporosis (67). Consequently, complementary treatments are being increasingly investigated, among which acupuncture has emerged as one of the most rapidly growing modalities. The pathogenesis of perimenopausal obesity involves complex mechanisms. Currently, it is known that acupuncture ameliorates obesity and perimenopausal symptoms through multi-level, multi-system, and multi-target synergistic mechanisms; however, the precise pathways remain incompletely elucidated. Furthermore, therapeutic efficacy may vary depending on acupoint selection, combination strategies, and stimulation parameters, aspects whose underlying mechanisms require further characterization.

This review critically evaluates key advancements in acupuncture-based interventions for perimenopausal obesity, which have informed current clinical approaches. The analysis encompassed electroacupuncture, acupoint catgut embedding, warm needle acupuncture, and moxibustion, with comparisons drawn against other acupuncture treatments, drug therapy, sham acupoint, or blank controls. The findings demonstrate that acupoint catgut embedding was particularly effective in improving BMI, body weight, estradiol levels, and perimenopausal symptoms compared with control groups. Warm needle acupuncture showed advantages in reducing body fat percentage. Electroacupuncture reduced WC but did not significantly improve BMI. Acupoint catgut embedding and warm needle acupuncture were shown to regulated serum sex hormones and blood lipid levels. The evidence indicates distinct therapeutic advantages among different acupuncture methods for managing perimenopausal obesity. To our knowledge, this study represents the first comprehensive network meta-analysis comparing the effects of various acupuncture methods for this condition, providing valuable evidence to support clinical decision-making in acupuncture protocol selection.

This review has several limitations: (1) Only one study used blinding, and most omitted details on randomization and allocation concealment, increasing bias risk. (2) The substantial heterogeneity among perimenopausal women, characterized by variations in obesity severity, physiological status, and hormonal fluctuations, may introduce potential performance bias in the implementation of interventions. The limited sample size of eligible studies precluded comprehensive subgroup analyses to evaluate how these factors affect treatment efficacy. (3) The current evidence for acupuncture in managing perimenopausal obesity remains relatively scarce, particularly regarding long-term therapeutic outcomes. The present findings are limited to short-term evidence, whereas the long-term sustainability and safety of acupuncture for this specific condition have yet to be established. (4) All included studies were single-center investigations, which limits the methodological robustness and generalizability of the findings due to the lack of multicenter validation. Future research should prioritize rigorously designed, large-scale randomized controlled trials to validate these preliminary observations.

## 5 Conclusion

In conclusion, our study demonstrated the efficacy of acupuncture-related interventions in improving multiple outcomes in perimenopausal women, including obesity indicators, perimenopausal symptoms, serum sex hormone levels, and lipid levels. Several acupuncture-related therapies may demonstrate superior efficacy compared to WM or herbal medicine HM in managing perimenopausal obesity. Furthermore, ACE emerged as the most effective intervention for reducing BMI and body weight and for improving the Kupperman Index. EA was most effective in reducing waist circumference, while WNA was ranked highest for reducing body fat percentage. These approaches could be considered as alternative or adjunctive treatment options in clinical practice. The selection of specific acupuncture methods should be individualized according to patients’ clinical requirements and actual conditions. However, these preliminary findings require verification through more multicenter, large-sample, randomized controlled clinical trialsin the future to establish more definitive evidence.

## Figures and Tables

**Table:** Glossary

NMA	Network Meta-Analysis
RCTs	Randomized Clinical Trials
GRADE	Grading of Recommendations, Assessment, Development, and Evaluation
BMI	Body Mass Index
WC	Waist Circumference
E_2_	Estradiol
LH	Luteinizing Hormone
FSH	Follicle-Stimulating Hormone
TG	Triglyceride
TC	Total Cholesterol
HDL	High-Density Lipoprotein
LDL	Low-Density Lipoprotein
MD	Weighted Mean Difference
CI	Confidence Interval
SUCRA	Surface Under the Cumulative Ranking Curve
ACE	Acupoint Catgut Embedding
EA	Electroacupuncture
WNA	Warm Needle Acupuncture
Moxa	Moxibustion
SA	Sham acupoint catgut embedding
HM	Traditional Chinese herbal medicine
WM	Western Medicine
None	No treatment
TCM	Traditional Chinese Medicine
MHT	Menopausal Hormone Therapy
AMPK	Adenosine 5’-Monophosphate (AMP)-activated Protein Kinase

## Data Availability

The original contributions presented in the study are included in the article/Supplementary material, further inquiries can be directed to the corresponding authors.
