# Supplementary material for: Comparison of efficacy of acupuncture-related therapy in the treatment of perimenopausal obesity: a network meta-analysis of randomized controlled trials
Source: Front Med (Lausanne). 2025 Nov 25;12:1642421. doi: 10.3389/fmed.2025.1642421 (PMC12685897; doi:10.3389/fmed.2025.1642421)
Supplement: Supplementary file 1 [file Supplementary_file_1.docx]

Supplement S1. Search strategies used in each database and the results

**Medline via PubMed**

|  | Searches | Results |
| --- | --- | --- |
| #1 | Obesity[MeSH Terms] OR Overweight[MeSH Terms] OR Obesity[Title/Abstract] OR Overweight[Title/Abstract] OR obese[Title/Abstract] OR observation[Title/Abstract] | 833,031 |
| #2 | Menopause[MeSH Terms] OR Postmenopause[MeSH Terms] OR Perimenopause[MeSH Terms] OR menopause[Title/Abstract] OR postmenopause[Title/Abstract] OR perimenopause[Title/Abstract] OR menopause-related[Title/Abstract] OR Post-Menopause[Title/Abstract] OR menopausal[Title/Abstract] OR Postmenopausal[Title/Abstract] | 121,265 |
| #3 | Acupuncture[MeSH Terms] OR “Acupuncture Therapy”[MeSH Terms] OR Auriculotherapy[MeSH Terms] OR “Acupuncture, Ear”[MeSH Terms] OR “auricular point”[Title/Abstract] OR Auriculotherapy[MeSH Terms] OR Acupressure[MeSH Terms] OR Electroacupuncture[MeSH Terms] OR “Acupuncture Points”[MeSH Terms] OR acupuncture[Title/Abstract] OR acupunctural[Title/Abstract] OR acupressure[Title/Abstract] OR acupoint*[Title/Abstract] OR acupoint[Title/Abstract] OR “trigger point”[Title/Abstract] OR “dry needling”[Title/Abstract] OR “ear acupuncture”[Title/Abstract] OR electroacupuncture[Title/Abstract] OR electro-acupuncture[Title/Abstract] OR pharmacopuncture[Title/Abstract] OR pharmaco-acupuncture[Title/Abstract] OR “Bee Venoms”[MeSH Terms] OR “bee venom*”[Title/Abstract] OR “acupoint injection”[Title/Abstract] OR Moxibustion[MeSH Terms] OR Moxibustion[Title/Abstract] OR moxa[Title/Abstract] OR “warm needling”[Title/Abstract] OR Meridians[MeSH Terms] OR “thread embedding”[Title/Abstract] OR “catgut embedding”[Title/Abstract] OR “needle embedding”[Title/Abstract] OR acupotomy[Title/Abstract] OR “needle knife”[Title/Abstract] OR thread-embedding[Title/Abstract] OR “thread embedding”[Title/Abstract] | 50,583 |
| #4 | #1 AND #2 AND #3 | 41 |

**EMBASE via Elsevier**

|  | Searches | Results |
| --- | --- | --- |
| #1 | 'obesity'/exp OR 'obesity':ab,ti OR 'overweight':ab,ti OR 'obese':ab,ti OR 'observation':ab,ti | 1,347,512 |
| #2 | 'menopause'/exp OR 'menopause':ab,ti OR 'postmenopause':ab,ti OR 'perimenopause':ab,ti OR 'menopause-related':ab,ti OR 'post-menopause':ab,ti OR 'menopausal':ab,ti OR 'postmenopausal':ab,ti | 172,199 |
| #3 | 'acupuncture'/exp OR 'acupuncture':ab,ti OR 'acupuncture therapy':ab,ti OR 'auriculotherapy':ab,ti OR 'acupuncture, ear':ab,ti OR 'auricular point':ab,ti OR 'acupressure':ab,ti OR 'acupuncture points':ab,ti OR 'acupunctural':ab,ti OR 'acupoint':ab,ti OR 'trigger point':ab,ti OR 'dry needling':ab,ti OR 'ear acupuncture':ab,ti OR 'electroacupuncture':ab,ti OR 'pharmacopuncture':ab,ti OR 'pharnacoacupuncture':ab,ti OR 'bee venoms':ab,ti OR 'bee venom':ab,ti OR 'acupoint injection':ab,ti OR 'moxibustion':ab,ti OR 'moxa':ab,ti OR 'warm needling':ab,ti OR 'meridians':ab,ti OR 'at embedding':ab,ti OR 'needle embedding':ab,ti OR 'acupotomy':ab,ti OR 'needle knife':ab,ti OR 'thread-embedding':ab,ti OR 'thread embedding':ab,ti | 73,589 |
| #4 | #1 AND #2 AND #3 | 53 |

WOS

|  | Searches | Results |
| --- | --- | --- |
| #1 | ((TS=("Obesity" OR "Overweight" OR "obese" OR "observation")) AND TS=("Menopause" OR "Postmenopause" OR "Perimenopause" OR "menopause-related" OR "Post-Menopause" OR "menopausal" OR "Postmenopausal")) AND TS=("Acupuncture" OR "Acupuncture Therapy" OR "Auriculotherapy" OR "Acupuncture, Ear" OR “auricular point" OR "Acupressure" OR "Electroacupuncture" OR “Acupuncture Points" OR "acupunctural" OR "acupoint" OR "trigger point" OR "dry needling" OR "ear acupuncture" OR "electroacupuncture" OR "pharmacopuncture" OR "pharnacoacupuncture" OR "Bee Venoms" OR "bee venom" OR "acupoint injection" OR "Moxibustion" OR "moxa" OR "warm needling" OR "Meridians" OR "thread embedding" OR "at embedding" OR "needle embedding" OR "acupotomy" OR "needle knife" OR "thread-embedding" OR "thread embedding") | 62 |

Cochrane

|  | Searches | Results |
| --- | --- | --- |
| #1 | MeSH descriptor: [Obesity] explode all trees | 53494 |
| #2 | ("Obesity" OR "Overweight" OR "obese" OR "observation"):ti,ab,kw | 113937 |
| #3 | MeSH descriptor: [Menopause] explode all trees | 8944 |
| #4 | ("Menopause" OR "Postmenopause" OR "Perimenopause" OR "menopause-related" OR "Post-Menopause" OR "menopausal" OR "Postmenopausal"):ti,ab,kw | 31123 |
| #5 | MeSH descriptor: [Acupuncture] explode all trees | 21878 |
| #6 | ("Acupuncture" OR "Acupuncture Therapy" OR "Auriculotherapy" OR "Acupuncture, Ear" OR “auricular point" OR "Acupressure" OR "Electroacupuncture" OR “Acupuncture Points" OR "acupunctural" OR "acupoint" OR "trigger point" OR "dry needling" OR "ear acupuncture" OR "electroacupuncture" OR "pharmacopuncture" OR "pharnacoacupuncture" OR "Bee Venoms" OR "bee venom" OR "acupoint injection" OR "Moxibustion" OR "moxa" OR "warm needling" OR "Meridians" OR "thread embedding" OR "at embedding" OR "needle embedding" OR "acupotomy" OR "needle knife" OR "thread-embedding" OR "thread embedding"):ti,ab,kw | 28061 |
| #7 | #1 OR #2 | 117520 |
| #8 | #3 OR #4 | 31488 |
| #9 | #5 OR #6 | 29225 |
| #10 | #7 AND #8 AND #9 | 67 |

CNKI

|  | Searches | Results |
| --- | --- | --- |
| #1 | (TKA=肥胖 + 超重) AND (TKA= 围绝经期 + 更年期 + 绝经后 + 绝经) AND (TKA= 针灸 + 针 + 针刺 + 电针 + 火针+ 温针 + 蜂针 + 耳针 + 艾灸 + 穴位 + 激光 + 穴位注射 + 穴位埋线 + 灸 + 耳穴 + 针刀 + 体针 + 子午流注) | 91 |

Wanfang data

|  | Searches | Results |
| --- | --- | --- |
| #1 | (主题=肥胖 OR 超重) AND (主题=围绝经期 OR 更年期 OR 绝经后 OR 绝经) AND (主题=针灸 OR 针 OR 针刺 OR 电针 OR 火针OR 温针 OR 蜂针 OR 耳针 OR 艾灸 OR 穴位 OR 激光 OR 穴位注射 OR 穴位埋线 OR 灸 OR 耳穴 OR 针刀 OR 体针 OR 子午流注) | 265 |

CQVIP

|  | Searches | Results |
| --- | --- | --- |
| #1 | (U=肥胖 OR 超重) AND (U=围绝经期 OR 更年期 OR 绝经后 OR 绝经) AND (U=针灸 OR 针 OR 针刺 OR 电针 OR 火针OR 温针 OR 蜂针 OR 耳针 OR 艾灸 OR 穴位 OR 激光 OR 穴位注射 OR 穴位埋线 OR 灸 OR 耳穴 OR 针刀 OR 体针 OR 子午流注) | 66 |

Sinomed

|  | Searches | Results |
| --- | --- | --- |
| #1 | ("肥胖"[常用字段:智能] OR "体重"[常用字段:智能]) OR ((("肥胖症"[不加权:扩展] OR "肥胖, 腹部"[不加权:扩展] OR "肥胖管理"[不加权:扩展]) OR "体重"[不加权:扩展])) | 237110 |
| #2 | ("更年期"[常用字段:智能] OR "绝经"[常用字段:智能] OR "围绝经期"[常用字段:智能] OR "绝经后"[常用字段:智能]) OR (("更年期"[不加权:扩展] OR "绝经后期"[不加权:扩展] OR "更年期综合征"[不加权:扩展]) OR "绝经期"[不加权:扩展] OR "围绝经期"[不加权:扩展] OR "绝经前期"[不加权:扩展]) | 52250 |
| #3 | ("针刀"[常用字段:智能] OR "针灸"[常用字段:智能] OR "针刺"[常用字段:智能] OR "电针"[常用字段:智能] OR "火针"[常用字段:智能] OR "耳针"[常用字段:智能] OR "温针"[常用字段:智能] OR "穴位注射"[常用字段:智能] OR "穴位埋线"[常用字段:智能]) OR ((((((((("针灸疗法"[不加权:扩展] OR "温针疗法"[不加权:扩展] OR "微波针刺疗法"[不加权:扩展] OR "针灸疗法"[不加权:扩展]) OR "穴, 阿是"[不加权:扩展] OR "针刺穴位"[不加权:扩展] OR "雷火灸疗法"[不加权:扩展]) OR "针刺"[不加权:扩展] OR "激光针刺疗法"[不加权:扩展]) OR "针刺疗法"[不加权:扩展] OR "超声波针刺疗法"[不加权:扩展] OR "激光针刺疗法"[不加权:扩展]) OR "微针疗法"[不加权:扩展]) OR "灸法"[不加权:扩展]) OR "水针疗法"[不加权:扩展]) OR "穴位按压"[不加权:扩展] OR "穴位贴敷疗法"[不加权:扩展]) OR "穴位埋线疗法"[不加权:扩展]) | 966054 |
| #3 | #1 AND #2 AND #3 | 148 |
