# Supplementary material for: Comparison of efficacy of acupuncture-related therapy in the treatment of perimenopausal obesity: a network meta-analysis of randomized controlled trials
Source: Front Med (Lausanne). 2025 Nov 25;12:1642421. doi: 10.3389/fmed.2025.1642421 (PMC12685897; doi:10.3389/fmed.2025.1642421)
Supplement: Supplementary file 2 [file Supplementary_file_2.docx]

**Supplement S2.**

**1.Baseline characteristics of studies included in the meta-analysis**

**2.Details of acupuncture therapies used in the included studies**

**3.Details of herbal medicine used in the included studies**

**1.Baseline characteristics of studies included in the meta-analysis**

| Study | Treatment | Concurrent therapy | Sample  size | Age(mean± SD or range) | Mean disease  duration,months(mean ± SD or range) | Follow-up(days) | Number of  acupuneture treatments | Outcome | Adverse events |
| --- | --- | --- | --- | --- | --- | --- | --- | --- | --- |
| Du  2011 | embedding therapy | Diet control | 32 | 50.3±3.0 | 10.89±4.81 | - | 1 time in every 2 weeks, 6 times as a course, and 1 course in total. | 1.Weight  2.BMI  3.Waist circumference  4.Kuppermanscale | Undocumented |
|  | No treatment |  | 32 | 49.8±3.2 | 9.02±4.29 | - | - | - | - |
| Lin2010 | Modulated middle-frequency electrical stimulation | None | 20 | 54.05±3.44 | - | - | electrical stimulation treatment for 20 min twice a week for 12 consecutive weeks | 1.Weight  2.waist-circumference  3.hip-circumference  4.BMI  5.percentage of lean muscle mass  6.Meridian System | Undocumented |
|  | No intervention |  | 21 | 56.05±3.11 | - | - | - | - | - |
| Chien2009 | Transcutaneous Electrical Stimulation | None | 20 | 57.0 (49.0, 63.0) | - | - | 30 min twice per week for 12 weeks | 1.Weight  2.waist -circumference  3.hip-circumference  4.WHR  5.BMI  6.percentage of lean muscle mass  7.HRV（HR、SDNN、RMSSD、To t a l p o w e r、LF、HF、LFnu、HFnu、LF/HF ratio） | Undocumented |
|  | No intervention |  | 20 | 52.5 (48.0, 61.0) | - | - | - | - | - |
| Chen2015 | catgut implantation at acupoint | oral  administration of Six Ingredient Rehmannia Pill | 30 | 51.27±3.473 | - | - | treatment for 1 time in 1 weeks, 4 weeks for 1 courses of treatment, a  total of 2 courses of treatment | 1.FSH  2.LH  3.E2  4.Weight  5.BMI  6.waist -circumference  7.Kupperman scale | Undocumented |
|  | No intervention |  | 30 | 49.77±2.849 | - | - | 8 pills each time, 3 times a day, 4 weeks as 1 course  of treatment, a total of 2 the course of treatment. | - | - |
| Deng2021 | the acupoint catgut embedding | Dietary adjustment | 30 | 49.5±2.5 | - | - | once a week for 3 consecutive weeks,rest for 1 week,for 1 course of treatment,3 course in total | 1.Weight  2.BMI  3.Abdomen-circumference  4.Kupperman scale  5.E2  6.LH  7.FSH. | Undocumented |
|  | No intervention |  | 30 | 50.2±2.8 | - | - | - | - | - |
| Du2020 | the acupoint catgut embedding | Diet and exercise guidance | 28 | 45.42±2.75 | - | 24 weeks | once every 2 weeks,8 times in total. | 1.Weight  2.BMI  3.Waist-circumference  4.Hip-circumference  5.WHR  6.TC  7.TG  8.HDL-C,LDL-C  9.AMH  10.SF-36scale | Undocumented |
|  | No intervention |  | 26 | 44.23±2.92 | - | 24 weeks | once every 2 weeks,8 times in total. | - | - |
| Huang2021 | Moxibustion for regulating Yin and Yang | Diet and exercise guidance | 34 | ５４．５０±５．８２ | - | - | 3 times a week,12 weeks in total. | 1.Weight-circumference  2.hip circumference  3.abdomen circumference  4.ＩＷＱＯＬ－Ｌｉｔｅscale | Undocumented |
|  | No intervention |  | 32 | ５３．９１±６．２０ | - | 12 times | 12 weeks | - | - |
| Huang2018 | ordinary acupuncture combined with electro-acupuncture | lifestyle  intervention | 28 | 49.9±4.6 | - | - | three times a week for 8  weeks, a total of 24 treatments | 1.MENQOL  2.Weight  3.waist Circumference  4.WHtR  5.BMI  6.Dual Energy X-ray 7.Absorptiometry (DEXA) | Undocumented |
|  | No intervention |  | 25 | 51.5±4.4 | - | - | 8 weeks | - | - |
| Luo2022 | acupoint catgut embedding method with  dredging and regulating Belt Vessel | lifestyle intervention | 34 | 49.32±3.07 | - | - | once a  week, 4 weeks as a course of treatment, a total of 2 courses of observation and  treatment | 1.Waist circumference,  2.hip circumference,  3.waist-hip ratio  4.body weight,  5.BMI,  6.F%  7.subcutaneous fat 8.thickness and blood 9.pressure  10.SF-36  11.IWQOL-Lite  12.SES  13.HAD | 5 |
|  | No intervention |  | 34 | 49.29±3.21 | - | - | 8week | - | - |
| Meng2022 | Warm needle moxibustion and acupuncture | Dietary adjustment | 59 | 49.57±2.14 | 1.90±0.42 | - | 3 times a week,1 month for 1 course,3 course of treatment | 1.Weight  2.F%  3.BMI  4.Kupperman-scale  5.FSH  6.E2  7.TCMSS | Undocumented |
|  | No intervention |  | 59 | 49.61±2.13 | 1.93±0.42 | - | 1 month for 1 course,3 course of treatment | - |  |
| Yu2017 | the acupoint catgut embedding | routine western medication | 70 | 51． 07 ± 4． 96 | 7． 37 ± 3． 35 | - | Once every 2 weeks,3 times for a course of treatment,6 months in total. | 1.waist circumference  2.hip circumference  3.body weight  4.BMI  5.TCM-syndrome integral  6.The-endometrial-thicknes  7.quantity of mature vaginal cells  8.serum values of estradiol and progesterone  9.levels of LH and FSH | Undocumented |
|  | No intervention |  | 70 | 50． 38 ± 5． 42 | 7． 43 ± 3. 68 | - | 6 months | - | - |
| Zhuang2023 | acupuncture | traditional Chinese medicine | 25 | 40.50± 5.14 | 2.13±0.31 | - | 8 weeks | 1.Clinical-efficacy  2.physical condition  3.lipid index  4.the obesity rate  5.body fat percentage  6.body mass index  7.HDL  8.LDL  9.triglyceride levels | Undocumented |
|  | No intervention |  | 25 | 50.50±5.12 | 2.33±0.14 | - | 8 weeks | - | - |
| Zhang2018 | electroacupuncture (EA) plus auricular acupuncture | None | 31 | 49±3 | 19.80±6.22 | - | 3 times in a week,1 course for 4 weeks,3 consecutive courses of treatment,3 months in total. | 1.The total efficacy  2.Kupperman score  3.Weight  4.BMI  5.WHR  6.the level of SHGB | Undocumented |
|  | taking Nilestriol |  | 31 | 50±3 | 19.27± 4.87 | - | 1 course for 4 weeks,3 consecutive courses of treatment,3 months in total. | - | - |
| Zhang2008 | embedded needle | Dietary adjustment | 30 | 50.33±3.03 | 10.90±4.82 | - | Performed every two weeks,and 3months was considered one observational treatment period | 1.weight  2.Waist-circumference  3.BMI  4.kupperman scale  5.E2  6.LH  7.FSH | Undocumented |
|  | No intervention |  | 32 | 49.81±3.20 | 8.03±4.30 | - | - | - | - |
| Xu2012 | Take meridians buried line therapy | None | 30 | - | - | - | Once a week,period of treatment for march | 1.Weight  2.BMI  3.Waist-circumference  4.F%. | Undocumented |
|  | Take simple oral Chinese medicine |  | 30 | - | - | - | Everyday 1.for 1 month for one period of treatment | - | - |
| Wang2020 | the acupoint catgut embedding | None | 30 | 56.14±1.77 | - | 1 day before treatment and half year after treatment. | - | 1.E2  2.LH  3.BMI | Undocumented |
|  | Take medicine |  | 30 | 55.81±1.98 | - | - | - |  | - |
| Wang2023 | acupoint thread embedding | lifestyle intervention | 44 | 50±4 | - | - | once every 2 weeks for 8 weeks (4 times in total) | 1.BMI  2.waist circumference  3.hip circumference  4.body mass,  5.modified Kupperman scale  6.insomnia severity index (ISI) score  7.self-rating anxiety scale (SAS) score  8.self-rating depression scale (SDS) score | 4 |
|  | No intervention |  | 43 | 49±4 | - | - | 8weeks | - | - |
| Sheng2020 | electroacupuncture | lifestyle intervention | 37 | 49.90±4.6 | - | - | Once every other day,three times a week,8 weeksin total. | 1.Weight  2.BMI  3.waist circumference  4.Hip-circumference  5.waist height ratio，(WHtR)  6.waist to hip ratio，(WHR)  7.menopause rating scale(MRS)  8.menopause-specific 9.quality of life(MENQOL)  10.TAG  11.Total-cholesterol  12.LDl  13.HDL  14.FINS  15.Glu,testos-terone(TT)  16.E2  17.homeostasis model assessment-insulin resistance(HOMA-IR) | Undocumented |
|  | No intervention |  | 36 | 51.50±4.4 | - | - | 8 weeks |  | - |
| Nie 2018 | the acupoint catgut embedding | Diet intervention | 18 | 51．07±8．88 | - | - | Once every 2 weeks,12 weeks in total | 1.Waist-circumference  2.Weight  3.BMI  4.Kupperman scale | Undocumented |
|  | No intervention |  | 22 | 50．38±5．41 | - | - | 12 weeks |  |  |
| Lu 2020 | the acupoint catgut embedding | Take oral medicine | 53 | 52. 0 ±  4. 1 | 8. 6± 1. 8 | - | Once every 2 weeks,3 times,1 course,12weeks in total. | 1.Waist circumference  2.Hip-circumference  3.Weight  4.BMI  5.E2  6.FSH  7.LH  8.MENQOL score | 4 |
|  | No intervention |  | 53 | 51. 2±4. 6 | 8. 9 ± 1. 6 | - | 12weeks | - | 7 |
| M.M. ELSAYED2022 | Laser biostimulation | a low-calorie diet | 30 | 66.10 ± 4.56  （60.00-75.00 ） | - | - | experimental group received LB and LCD  (including 50-60% carbohy- drates, 15-20%  protein, 20-35% fat, and 25 g of fiber/day plus a  restriction of 500-1000 kcal/d)，12weeks. | 1.Glucose  2.insulin levels  3.HOMA-IR index  4.the inflammatory 5.biomarkers (WBCs  count, lympho- cytes, ESR, and CRP levels)  6.A depression assessment scale (HAMD-17-  item scale)  7.BMI  8.Weight  9.height | Undocumented |
|  | No intervention |  | 30 | 67.12 ± 5.06  （60.00-75.00） | - | - | the control  group followed the same diet program only for  12 weeks. | - | - |
| PIOTR WOZNIAK2003 | one cycle of laser acupuncture | a low-calorie diet | 38 | 54.92 ( ±3.81) | - | - | 6 months | 1.BMI  2.Body-weight  3.WHR  4.FSH  5.E2  6.WB  7.Fasting glucose  8.Total cholesterol  9.HDL cholesterol  10.LDL cholesterol  11.Triglycerides | Undocumented |
|  | No intervention |  | 36 | 55.19 ( ±4.14) | - | - | 6 months | - | - |

**2.Details of acupuncture therapies used in the included studies**

| **Study ID** | **Stimulation**  **method** | **Acupoint** | **Needle retention or**  **acupressure time** | **Frequency** | **Treatment**  **duration** | **Total sessions** | **Others** |
| --- | --- | --- | --- | --- | --- | --- | --- |
| Du  2011 | Catgut embedding therapy | *sthenia heat of stomach and intestine type: ST 44, ST 36， ST 37, ( ST 40, ST 12, SP 6;  *dampness inhibition due to spleen deficiency type: BL 20, ST37, ST 12, ST 36, SP 6;  *liver *qi* stagnation type: LR 3, ST 12, ST 36, BL 18, LR 14, SP 6; *kidney *yang* deficiency type: CV 4, ST 36, BL 23, BL 20, CV 6, SP 6;  *kidney *yin* deficiency type: CV 4, BL 18, ST 36, BL 23, SP 6, KI 3, LR 3。 | Not recorded | once every 2 weeks | 12 weeks | 6 | Use medical catgut as materials.  medical catgut was cut into small  pieces by 1 cm in length |
| Lin  2010 | Electrical Acupoint Stimulation | ST 36, SP 6 | 20 min | twice a week | 12 weeks | 24 | None |
| Chien  2011 | transcutaneous electric acupoint stimulations (TEAS) | ST 36, SP 6 | 30 min | twice a week | 12 weeks | 24 | maximum output current 50 mA, output voltage 0–25 V and wave width 125 μA;two different frequency modulations (FM) are mixed, the frequency of channel one set at 4000 Hz and channel two at 61 Hz |
| Lin  2010 | Electrical Acupoint Stimulation | ST 36, SP 6 | 20 min | twice a week | 12 weeks | 24 | None |
| Chien  2009 | Transcutaneous Electrical Stimulation | ST 36, SP 6 | 30 min | twice a week | 12 weeks | 24 | None |
| Chen  2016 | Catgut embedding therapy | BL 23, SP 6, CV 4, CV 6, ST 29, EX-CA 1, CV 12, ST 21, ST25,SP 15. | Not recorded | Once a week | 4 weeks | 4 | Six Ingredient Rehmannia Pill (Lanzhou foci Pharmaceutical Co., Ltd., the country medicine accurate Z62020664, 0.375g/ particle) 8 pills each time, 3 times a day, 4 weeks as 1 course of treatment, a total of 2 the course of treatment |
| Deng  2021 | Catgut embedding therapy | KI 19, ST 25, SP 15, ST 36, EX-CA 1 , SP 6.  *liver *qi* stagnation type:BL 18, GB 34,  *liver ddepression and spleen deficiency type: BL 18, BL 20,  *spleen-kidney yang deficiency type: BL 20, BL 23, KI 13  *liver-kidney yin deficiency type : BL 18, BL 23, SP 9,  *yin and yang deficiency type :BL 23, DU 4, CV 4 | Not recorded | Once a week | 12 weeks | 12 | None |
| Du  2020 | catgut embedding therapy | A Group:ST 25, BL 21, CV 12, ST 36, SJ 6  B Group:ST 40, BL 20, CV 9, ST 24, LI 11 | Not recorded | once every 2 weeks | 16 weeks | 8 | None |
| Huang  2021 | moxibustion | BL 20, BL 23,DU 4, CV 4, CV 8 | 20 min | Three times a week | 12 weeks | 36 | None |
| Huang  2018 | Electroacupuncture | CV 12, CV 10, CV 6 ,ST 25, SP 15, GB 26, SP 14,  *liver-kidney yin deficiency type :K 13, LR 3,SP 6, BL18, BL 21  *spleen-kidney yang deficiency type: CV 4, DU 4, ST 36, BL 23, BL 20,  *dampness inhibition due to spleen deficiency type: SP 4, SP 9, SP 8, SP 40,BL 20  *liver depression and spleen deficiency type: LI 4, LR 3, BL18,  *Stomach heat and dampness obstruction type: LI 11, SP 6, ST 44, SP 4, BL21, BL 20 | 10/20 min | Three times a week | 8 weeks | 24 | None |
| Luo  2022 | Catgut embedding therapy | GB 26, GB 27,, GB 28, CV 12, ST 24, ST 25, SP 15, CV 4, BL 23, BL 25, BL 22, ST 36, ST 40 | None | Once a week | 8 weeks | 8 | None |
| Meng  2022 | Warm acupuncture and moxibustion | CV 12, CV 9, ST 28, CV 4, SP 15, SP 14, ST 40, SP 9 | 10～15min | Three times a week | 3 months | 36 | None |
| Nie  2018 | Catgut embedding therapy | CV 12, BL 20, ST 25, ST 36, SP 6, ST 40. | None | once every 2 weeks | 12 weeks | 6 | None |
| Sheng  2022 | Electroacupuncture | CV 12, CV 6, CV 4,ST 25, SP 15,BL 20 ,BL 23  *Stomach heat stagnation in the spleen type: SP 6, ST 44  *Phlegm dampness and inteCVal accumulation type : SP 9, ST40  *liver *qi* stagnation type: LI 4, LR 3,  *spleen deficiency and stagnation type : ST 36, SP 3  *spleen-kidney yang deficiency type: DU 4, BL 62 | 30min | Three times a week | 8 weeks | 24 | None |
| Wang  2020 | Catgut embedding therapy | SP 15, CV 12, ST 25, BL 15, BL 18, BL 23, BL 20, DU 4 | None | once | once | 1 | None |
| Wang  2023 | Catgut embedding therapy | CV 13, CV 12, CV 10, CV 6, CV 4, CV 3, SP 15, ST 25, SP 9, ST 40  *Stomach and intestines heat type: LI 11, SJ 6  *Endogenous phlegm dampness type: CV 9 ,SP 9  *spleen deficiency and stagnation type : ST 36, BL 20  *spleen-kidney yang deficiency type: BL 20, BL 23 | None | once every 2 weeks | 8 weeks | 4 | None |
| Xu  2012 | Catgut embedding therapy | A Group: CV 4, ST 25, GB 26, BL 23, ST 36, ST 37  B Group: CV 6, SP 15, ST 28, BL 20, BL 52, ST 40 | None | Once a week | 3 months | 12 | Compared with Chinese Medicine |
| Lv  2020 | Catgut embedding therapy | ST 25, CV 12, ST 21, LR 8, ST 40, LR 3,CV 6,ST 36 , BL 20, BL 32 | None | once every 2 weeks | 12 weeks | 6 | Oral administration of mirtazapine tablets, once per day, starting at a dose of 15 mg, gradually increasing the dose to 30 mg, starting at a dose of 2 mg for nilestradiol tablets, supplemented with estrogen every 2 weeks, with a 12 week course of treatment |
| Yu  2017 | Catgut embedding therapy | ST 25, CV 12, ST 21,LR 8, ST 40, LR 3, CV 6, ST 36 | None | once every 2 weeks | 12 weeks | 6 | Oral administration of mirtazapine tablets, once per day, starting at a dose of 15 mg, gradually increasing the dose to 30 mg, starting at a dose of 2 mg for nilestradiol tablets, supplemented with estrogen every 2 weeks, with a 12 week course of treatment |
| Zhang  2008 | Catgut embedding therapy | *Stomach and intestines heat type: ST 44, ST 36, CV 12, ST 37, ST 25, SP 6  *dampness inhibition due to spleen deficiency type: BL 20,ST 37, ST 25, ST 36, SP 6, ST 40  *liver *qi* stagnation type: LR 3, ST 25, ST 36 ,BL 18, LR 14, SP 6  *kidney *yang* deficiency type: CV 4, ST 36, BL 23, BL 20, CV 6, SP 6  *kidney *yin* deficiency type:CV 4, BL 18, ST 36, ,BL 23, SP 6, LR 3 | None | once every 2 weeks | 12 weeks | 6 | None |
| Zhang  2018 | Electroacupuncture  Auricular acupressure | ST 25, SP 15, CV 12, CV 9, ST 24, CV 6, LI 11, HT 7, ST 36, ST 40, SP 6, KI 3, LR 3  Auricular points: Shen men , liver, endocrine | 30 min | Three times a week | 12 weeks | 36 | Oral Nilestriol tablets, 2 mg each time, taken every two weeks. After symptoms improve, the maintenance dose is 1 mg each time, and it is 1 for 4 weeks  One course of treatment, continuous treatment for three courses |
| Zhuang  2023 | Warm acupuncture and moxibustion | BL 23, CV 12, DU 4,CV 4, BL 58, SP 3, CV 2 | 25～30 min | once every other day | 8 weeks | 28 | Compared with Chinese Medicine |

1. **Details of herbal medicine used in the included studies**

| **Study ID** | **Name of herbal medicine** | **Dosage**  **form** | **Administr**  **ation**  **duration** | **Composition and dose of individual herb** | **Modifying components** | **Manufacturin**  **g company**  **(Chinese**  **patent**  **medicine)** |
| --- | --- | --- | --- | --- | --- | --- |
| Huang 2021 | modified Wendan-tang | Decoction | 12 weeks | Alismatis Rhizoma, Phyllostachyos Caulis in Taeniam, Pinelliae Tuber,  Polygalae Radix, Citri Unshius Pericarpium, Acori Graminei Rhizoma each  10 g, Glycyrrhizae Radix et Rhizoma 6 g, Zingiberis Rhizoma Recens 5  pieces, Zizyphi Fructus 3 g  (per day) | -edema: Benincasae  Exocarpium 30 g, Polyporus 10 g  -anorexia: Atractylodis  Rhizoma Alba, Codonopsis Pilosulae Radix each 10 g (per day) | Not applicable |
| Pang  2010 | -spleen deficiency with dampness  obstruction: modified Pingwei-san -stomach heat with dampness  obstruction: modified Xiehuang-san  -dual deficiency of spleen and kidney: modified Liujunzi-tang  -yin deficiency with internal heat: modified Qiju-Dihuang-hwan | Not  recorded | 60 days | Not recorded | None | Not applicable |
| Qin  2016 | modified Cangfu-Daotan-granule | Granule | 12 weeks | Atractylodis Rhizoma, Cyperi Rhizoma, Scutellariae Radix, Poria  Sclerotium, Curcumae Radix, Trichosanthis Semen, Angelicae Gigantis  Radix each 10 g, Pinelliae Tuber, Citri Unshius Pericarpium, Ponciri  Fructus Immaturus each 6 g, Amomi Fructus Rotundus, Magnoliae Cortex,  Coptidis Rhizoma each 3 g  (per 2/3~2 days according to the age) | None | Jiangfen  Jiangyin  Tianjiang  Pharmaceutical  Co., Ltd. |
| Shen  2001 | Jiangzhi-tablet | Tablet | 3 months | Rhei Radix et Rhizoma 6~15 g  (per day) | None | Not applicable |
| Song  2017 | Fangfeng-tongsheng-pill | Pill | 30 days | Saposhnikoviae Radix, Schizonepetae Spica, Menthae Herba, Ephedrae  Herba, Rhei Radix et Rhizoma,Natrii Sulfas, Gardeniae Fructus, Talcum,  Platycodonis Radix, Gypsum Fibrosum, Cnidii Rhizoma, Angelicae  Gigantis Radix, Paeoniae Radix, Scutellariae Radix, Forsythiae Fructus,  Glycyrrhizae Radix et Rhizoma, Atractylodis Rhizoma Alba | None | Beijing  Tongrentang  Technology  Development  Co., Ltd. |
| Wang 2019b | Yiqi-Jianpi-tang | Decoction | 3 months | Stigma Maydis 15 g, Astragali Radix, Crataegi Fructus each 12 g,  Pseudostellariae Radix 9 g, Citri Unshius Pericarpium, Salviae  Miltiorrhizae Radix, Pinelliae Tuber each 6 g  (per 1~1.5 days according to the age) | None | Not applicable |
| Wang  2021a | Yiqi-Jianpi-tang | Decoction | 1 month | Stigma Maydis 15 g, Astragali Radix, Crataegi Fructus each 12 g,  Pseudostellariae Radix 9 g, Citri Unshius Pericarpium, Salviae  Miltiorrhizae Radix, Pinelliae Tuber each 6 g  (per 1~1.5 days according to the age) | None | Not applicable |

| Xiao  2008 | (A) Jianfei oral liquid  (B) Fangfeng-tongsheng-pill | (A) Oral  liquid  (B) Pill | 3 months | (A) Cassiae Semen, Coicis Semen each 15 g, Poria Sclerotium 12 g,  Pharbitidis Semen, Nelumbinis Folium, Atractylodis Rhizoma, Atractylodis  Rhizoma Alba, Citri Unshius Pericarpium, Salviae Miltiorrhizae Radix,  Crataegi Fructus each 10 g, Pinelliae Tuber, Arecae Pericarpium each 8 g  (per 1/6~1/3 days according to the age)  (B) Not recorded | None | Not applicable |
| --- | --- | --- | --- | --- | --- | --- |
| Xing  2009 | Erchen-tang | Decoction | 2 months | Poria Sclerotium 10 g, Mume Fructus 9 g, Pinelliae Tuber, Citri Unshius  Pericarpium each 7 g, Zingiberis Rhizoma Recens, Glycyrrhizae Radix et  Rhizoma each 6 g  (per 2 days) | -food accumulation: Crataegi Fructus 9 g  -heavy dampness: Polygoni  Cuspidati Rhizoma et Radix 7 g  -spleen qi deficiency:  Codonopsis Pilosulae Radix 10 g  (per 2 days) | Not applicable |
| Xiong 2014 | Erchen-tang | Decoction | 20 days | Poria Sclerotium 10 g, Mume Fructus 9 g, Pinelliae Tuber, Citri Unshius  Pericarpium each 7 g, Zingiberis Rhizoma Recens, Glycyrrhizae Radix et  Rhizoma each 6 g  (per day) | None | Not applicable |
| Yang  2003 | Children's Jianfei-capsule | Capsule | 3 months | Pinelliae Tuber, Poria Sclerotium, Alismatis Rhizoma, Magnoliae Cortex | None | Yunnan  University of  Traditional  Chinese  Medicine  Pharmaceutical  Factory |
| Yang  2018 | modified Wendan-tang | Decoction | 12 weeks | Poria Sclerotium 15 g, Alismatis Rhizoma, Phyllostachyos Caulis in  Taeniam, Pinelliae Tuber, Polygalae Radix, Acori Graminei Rhizoma, Citri  Unshius Pericarpium each 10 g, Glycyrrhizae Radix et Rhizoma 6 g,  Zizyphi Fructus 3 g, Zingiberis Rhizoma Recens 5 pieces  (per day) | None | Not applicable |
| Yao  2019 | modified Erchen-tang | Decoction | 3 months | Poria Sclerotium, Pinelliae Tuber, Salviae Miltiorrhizae Radix, Nelumbinis  Folium, Ponciri Fructus Immaturus, Arecae Semen, Crataegi Fructus each 9  g, Citri Unshius Pericarpium 6 g, Glycyrrhizae Radix et Rhizoma 3 g  (per day) | None | Not applicable |
| Zhang 2020 | Erchen-tang | Decoction | 2 months | Poria Sclerotium 10 g, Mume Fructus 9 g, Pinelliae Tuber, Citri Unshius  Pericarpium each 7 g, Zingiberis Rhizoma Recens, Glycyrrhizae Radix et  Rhizoma each 6 g  (per day) | -food accumulation: Crataegi Fructus 9 g  -heavy dampness: Polygoni  Cuspidati Rhizoma et Radix 7 g  -spleen qi deficiency:  Codonopsis Pilosulae Radix 10 g  (per day) | Not applicable |

| Zhou  2016 | Fangfeng-tongsheng-san | Decoction | 3 months | Saposhnikoviae Radix, Schizonepetae Spica, Forsythiae Fructus, Menthae  Herba, Cnidii Rhizoma, Angelicae Gigantis Radix, Paeoniae Radix,  Atractylodis Rhizoma Alba, Gardeniae Fructus, Rhei Radix et Rhizoma,  Natrii Sulfas, Gypsum Fibrosum, Scutellariae Radix, Platycodonis Radix,  Glycyrrhizae Radix et Rhizoma, Talcum | None | Not applicable |
| --- | --- | --- | --- | --- | --- | --- |
| Xu  2012 | You gui wan + Shen ling bai zhu san | Decoction | 3 months | Aconite 10g, Cinnamomum 10g, Rehmannia 20g,Chinese Yam, 10g of Angelica sinensis 20g, Goji berries 15g, Eucommia ulmoides 15g, Cornus officinalis 10g, Codonopsis pilosula 15g, Poria cocos 10g, Atractylodes macrocephala 10g, Leng beans 20g, Yiren 20g, Lotus flesh 20g,Amomum villosum 5g, raw licorice 6g, Platycodon grandiflorum 10g,dried tangerine peel 5g, 5 slices of ginger. | None | Not applicable |
| Zhuang  2023 | Si jun zi-tang | Decoction | 8 weeks | PaoFuZi 6 g PaoJiang 6 g, Codonopsis pilosula 12 g, Atractylodes macrocephala 12 g, Poria cocos 12 g, Poria cocos 12 g, Zexie 12 g, roasted sweet Grass 6 g | Yam, lentils, cinnamon | Not applicable |
