# Supplementary material for: Comparison of efficacy of acupuncture-related therapy in the treatment of perimenopausal obesity: a network meta-analysis of randomized controlled trials
Source: Front Med (Lausanne). 2025 Nov 25;12:1642421. doi: 10.3389/fmed.2025.1642421 (PMC12685897; doi:10.3389/fmed.2025.1642421)

**Direct comparison of meta-analysis results**

| **Direct comparison of meta-analysis results** | | | | | | |
| --- | --- | --- | --- | --- | --- | --- |
| BMI | Comparison category | Number of studies | Heterogeneity | | Meta analysis results | |
|  |  |  | *I^2^* | *P* | MD/SMD, 95%CI | *P* |
|  | Acupoint catgut embedding vs No treatment | 9 | 0% | 0.97 | **-2.24 [-2.64, -1.84]** | **<0.00001** |
|  | Acupoint catgut embedding vs Western medicine | 1 | NA | NA | **-3.71 [-6.59, -0.83]** | **0.01** |
|  | Acupoint catgut embedding vs Sham Acupuncture | 1 | NA | NA | **-1.09 [-2.04, -0.14]** | **0.02** |
|  | Acupoint catgut embedding vs Chinese medicine | 1 | NA | NA | **-2.64 [-4.98, -0.30]** | **0.03** |
|  | Eletro-acupuncture vs No treatment | 2 | 0% | 0.7 | -0.76 [-1.62, 0.09] | 0.08 |
|  | Eletro-acupuncture vs Western medicine | 1 | NA | NA | **-1.42 [-2.37, -0.47]** | **0.003** |
|  | Warm needle vs No treatment | 2 | 0% | 0.48 | **-1.91 [-2.53, -1.28]** | **<0.00001** |
| Body weight | Acupoint catgut embedding vs No treatment | 9 | 57% | 0.02 | **-4.11 [-5.83, -2.39]** | **<0.00001** |
|  | Acupoint catgut embedding vs Chinese medicine | 1 | NA | NA | **-5.64 [-9.55, -1.73]** | **0.005** |
|  | Acupoint catgut embedding vs Sham Acupuncture | 1 | NA | NA | -2.66 [-5.54, 0.22] | 0.07 |
|  | Eletro-acupuncture vs No treatment | 2 | 0% | 0.74 | -0.70 [-3.10, 1.71] | 0.57 |
|  | Eletro-acupuncture vs Western medicine | 1 | NA | NA | **-2.80 [-5.39, -0.21]** | **0.03** |
|  | Moxibustion vs No treatment | 1 | NA | NA | **-3.31 [-6.35, -0.27]** | **0.03** |
| Waist circumference | Acupoint catgut embedding vs No treatment | 9 | 0% | 0.94 | **-3.43 [-4.00, -2.87]** | **<0.00001** |
|  | Acupoint catgut embedding vs Sham Acupuncture | 1 | NA | NA | **-4.12 [-6.15, -2.09]** | **<0.00001** |
|  | Acupoint catgut embedding vs Chinese medicine | 1 | NA | NA | **-7.64 [-11.78, -3.50]** | **0.0003** |
|  | Eletro-acupuncture vs No treatment | 2 | 0% | 0.74 | **-4.82 [-6.96, -2.68]** | **<0.0001** |
|  | Moxibustion vs No treatment | 1 | NA | NA | **-4.41 [-8.04, -0.78]** | **0.02** |
| Hip circumference | Acupoint catgut embedding vs No treatment | 4 | 0% | 0.47 | **-3.63 [-4.87, -2.38]** | **<0.00001** |
|  | Acupoint catgut embedding vs Sham Acupuncture | 1 | NA | NA | -1.08 [-3.48, 1.32] | 0.38 |
|  | Eletro-acupuncture vs No treatment | 1 | NA | NA | -1.86 [-3.94, 0.22] | 0.08 |
|  | Moxibustion vs No treatment | 1 | NA | NA | -1.29 [-3.35, 0.77] | 0.22 |
| Body fat rate | Acupoint catgut embedding vs No treatment | 1 | NA | NA | **-2.20 [-3.54, -0.86]** | **0.001** |
|  | Acupoint catgut embedding vs Chinese medicine | 1 | NA | NA | **-3.84 [-7.41, -0.27]** | **0.04** |
|  | Warm needle vs No treatment | 2 | 0% | 0.34 | **-3.71 [-4.94, -2.48]** | **<0.00001** |
| Kupperman | Acupoint catgut embedding vs No treatment | 5 | 42% | 0.14 | **-5.63 [-7.81, -3.44]** | **<0.00001** |
|  | Warm needle vs No treatment | 1 | NA | NA | **-3.79 [-5.14, -2.44]** | **<0.00001** |
| E^2^ | Acupoint catgut embedding vs No treatment | 5 | 86% | <0.0001 | **22.72[11.25, 34.18]** | **0.0001** |
| FSH | Acupoint catgut embedding vs No treatment | 5 | 73% | 0.006 | **-5.80[-7.94, -3.65]** | **<0.00001** |
| LH | Acupoint catgut embedding vs No treatment | 5 | 87% | <0.00001 | **-8.38[-11.44, -5.32]** | **<0.00001** |
| MENQOL | Eletro-acupuncture vs No treatment | 2 | 0% | 0.67 | **-31.36[-34.98, -27.74]** | **<0.00001** |
| Abbreviation：NA, Data unavailable; MD, Mean Difference; SMD, Standard Mean Difference; CI, Confidence interval. The bold font indicates that there was a statistically significant difference between the two treatments. | | | | | | |

**NMA**

1. body mass index


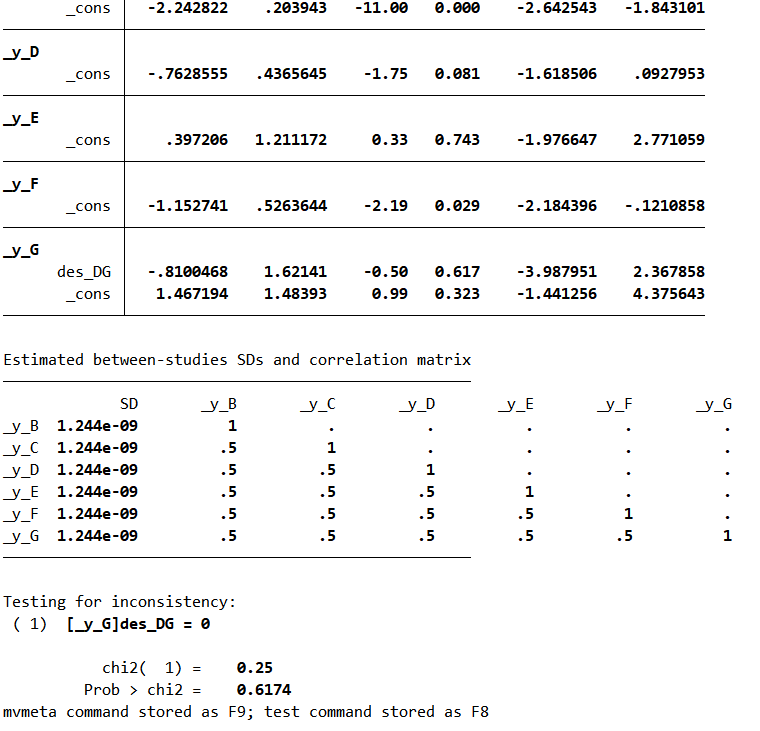


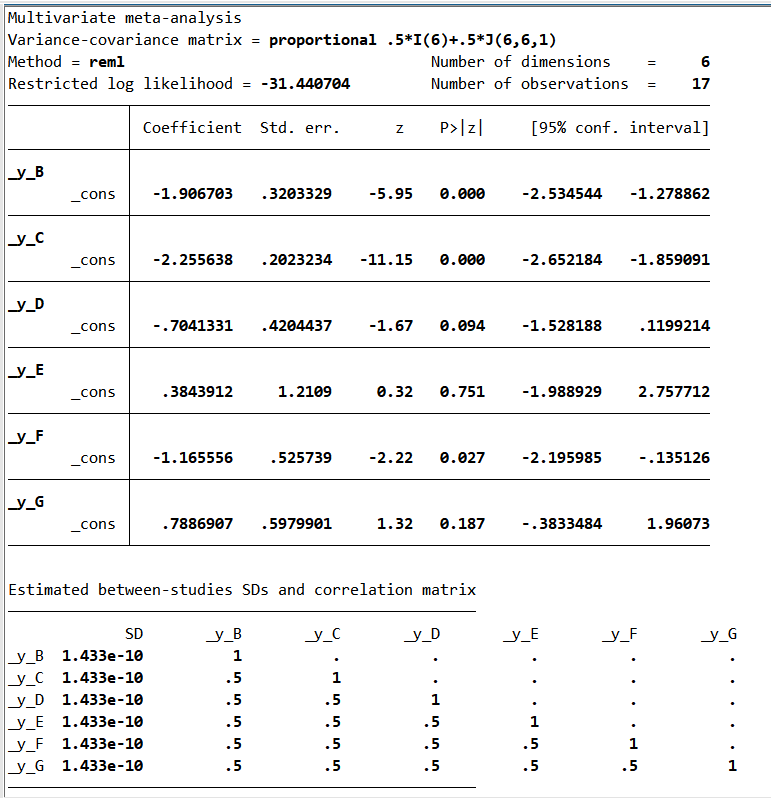


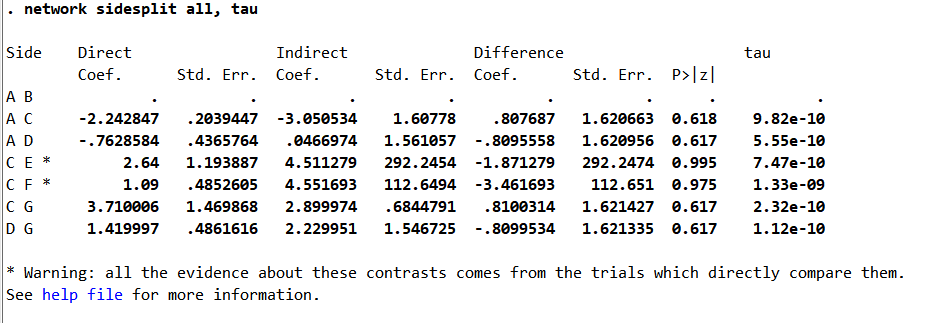


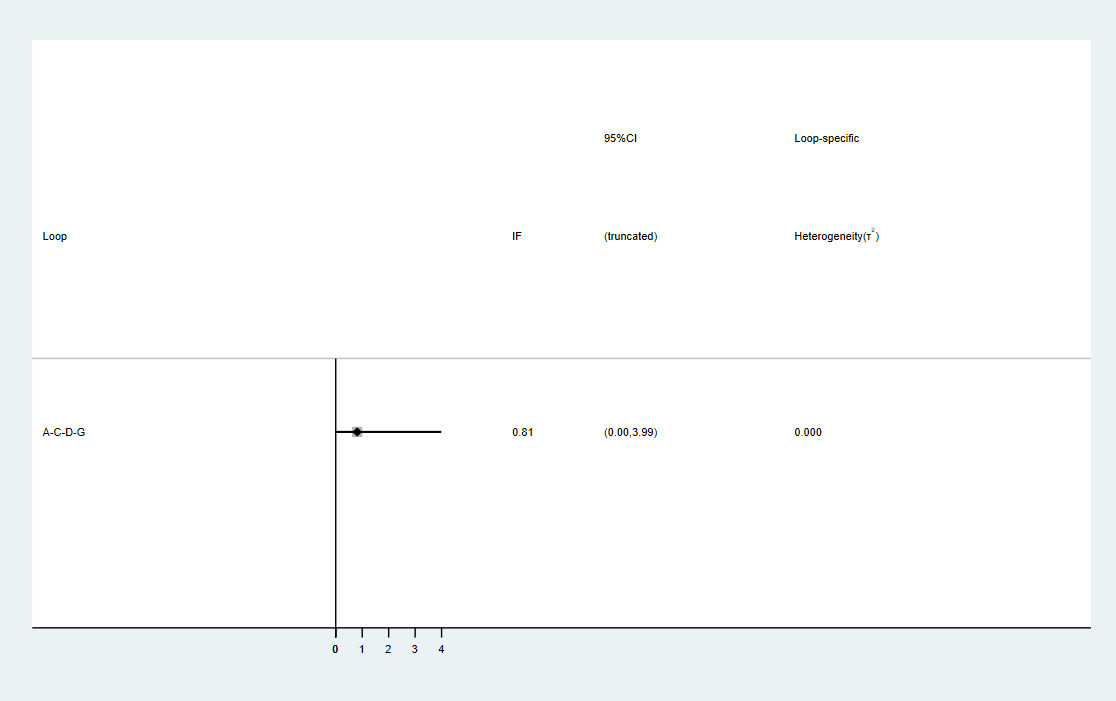


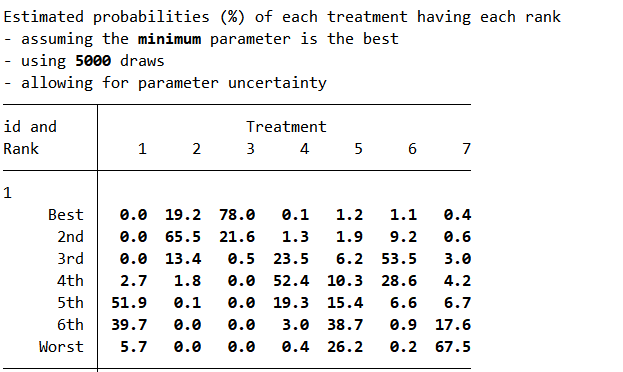


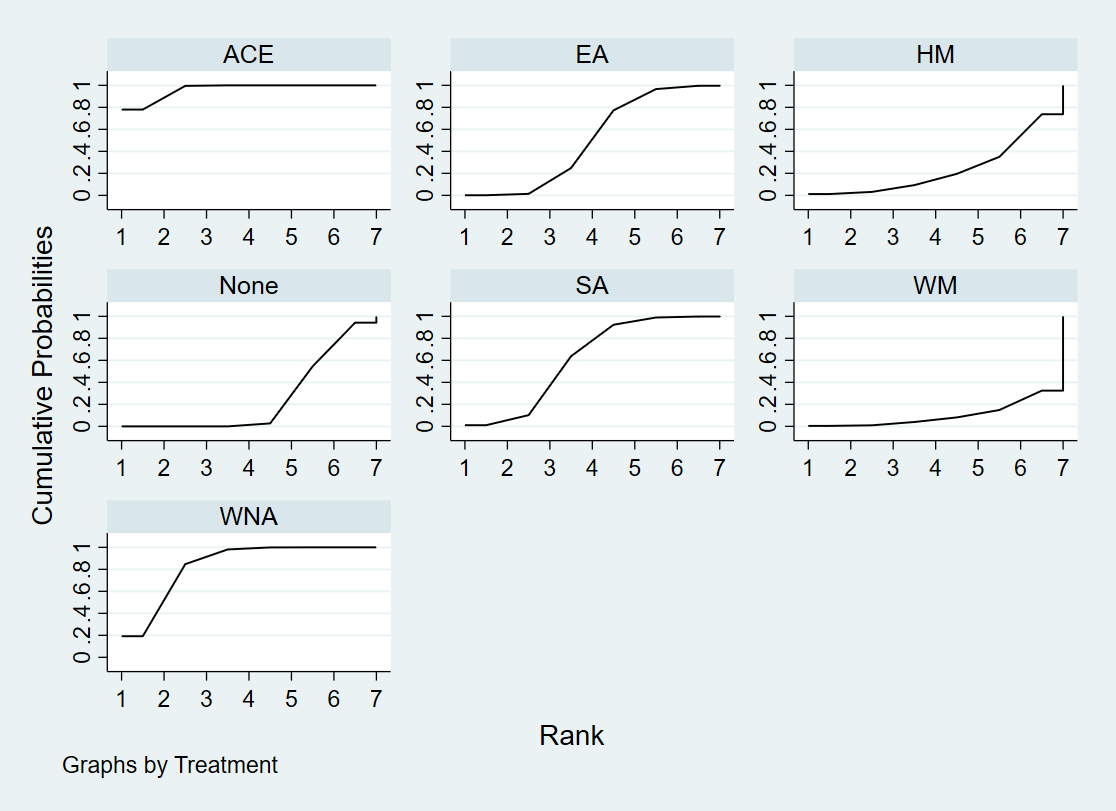


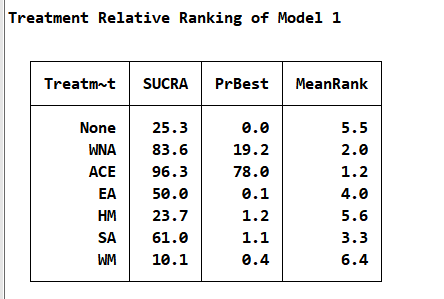


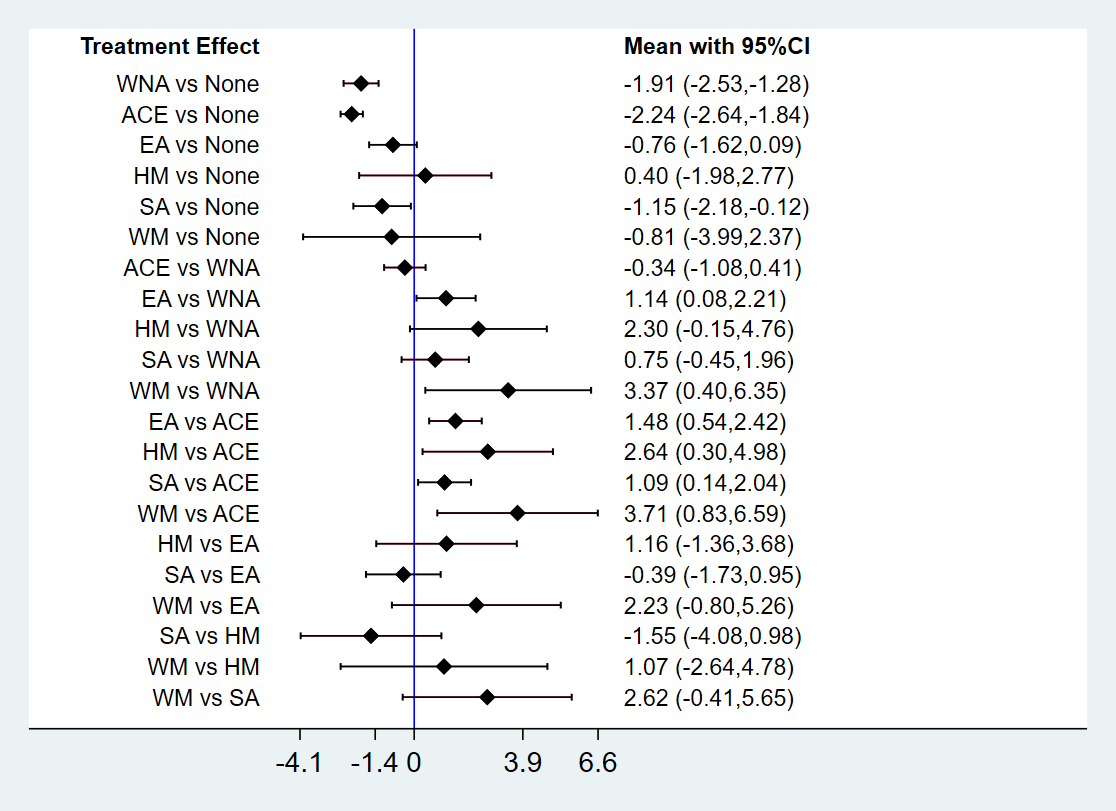


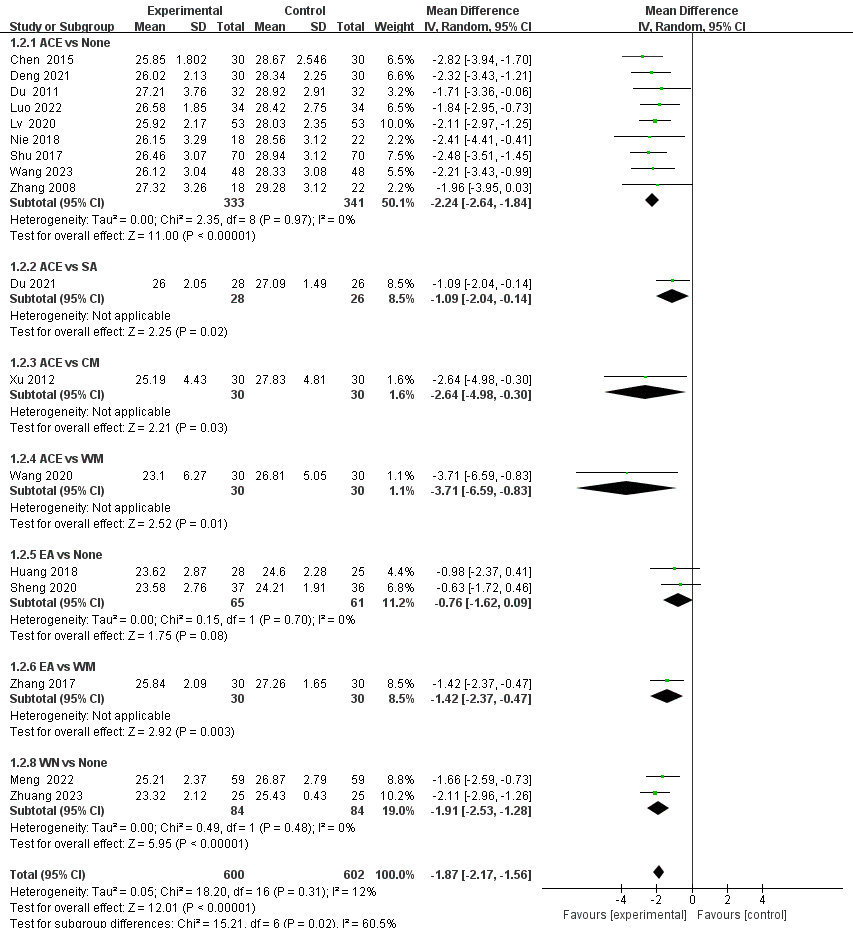


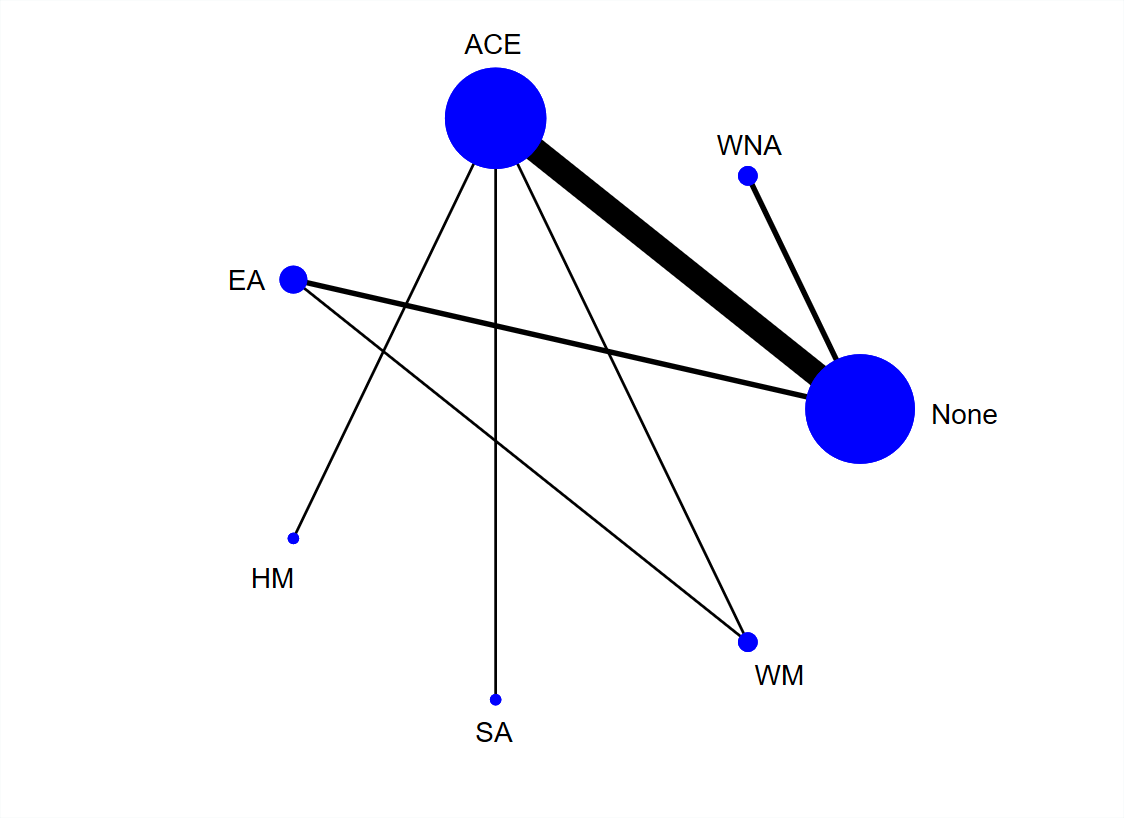


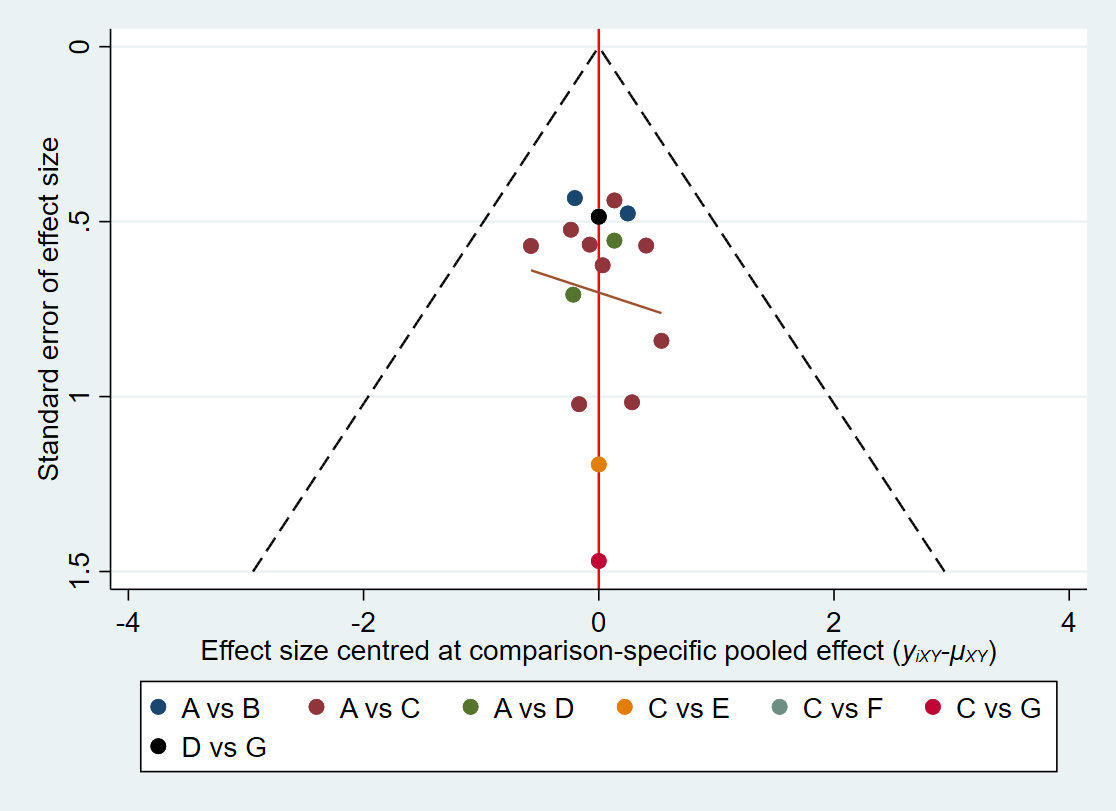


1. waist circumference


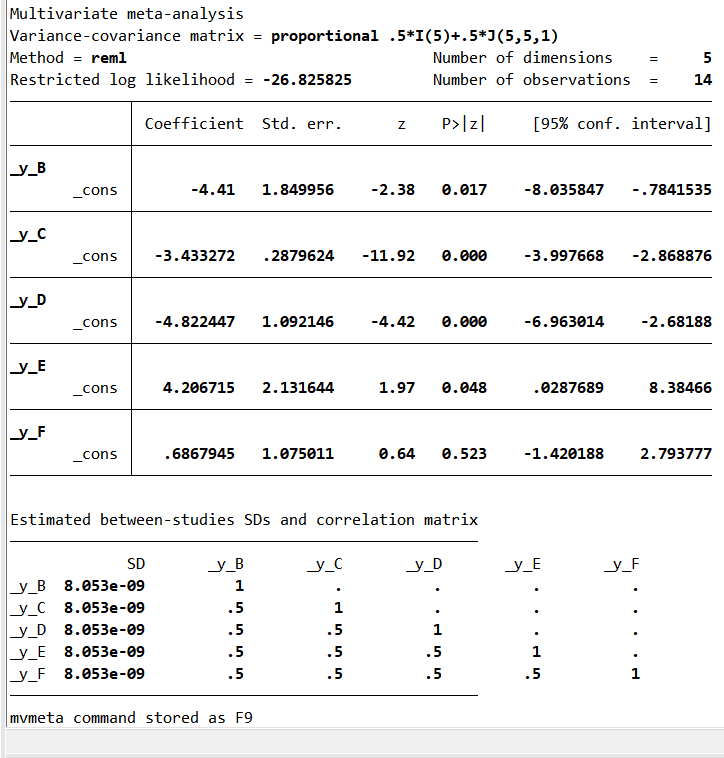


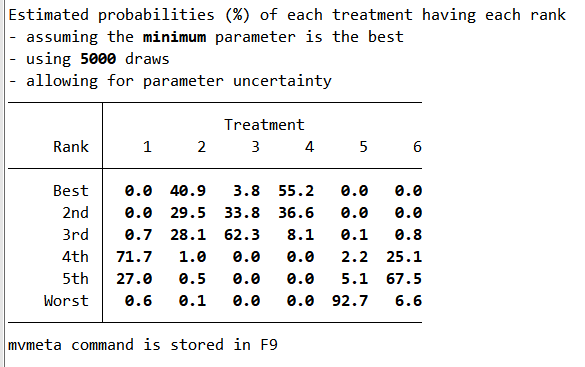


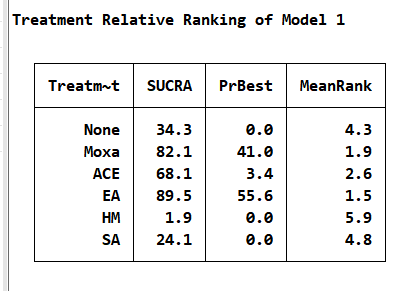


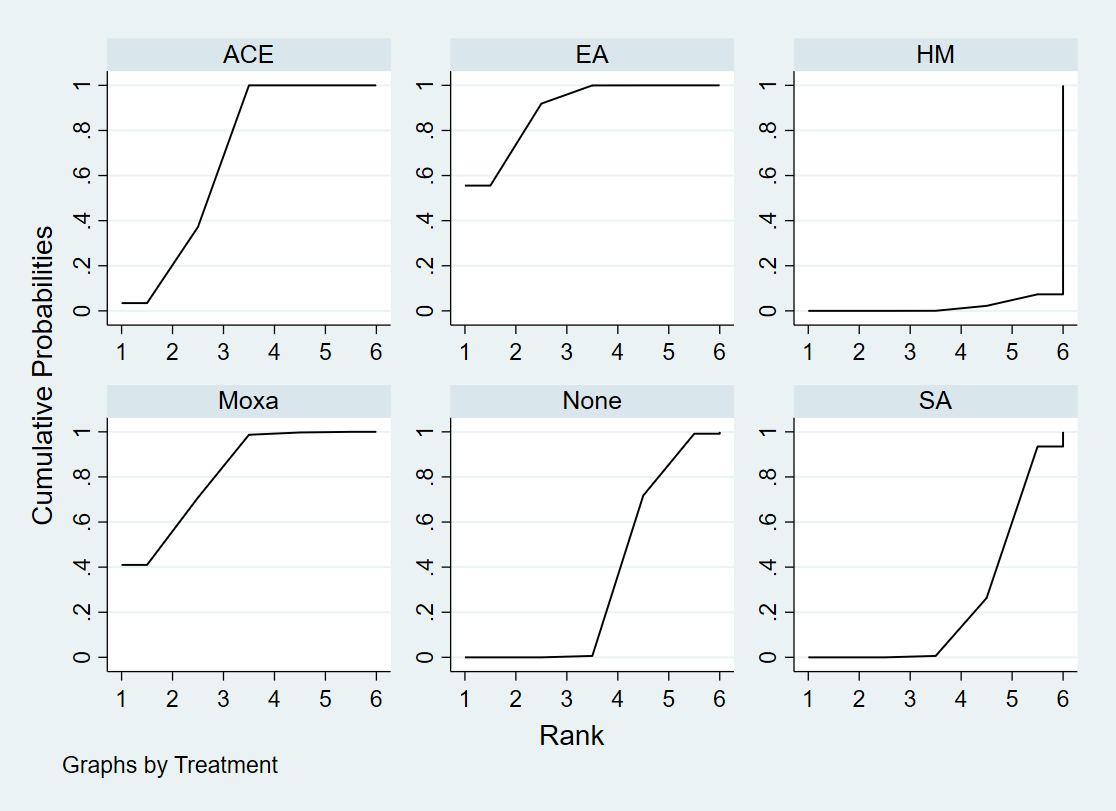


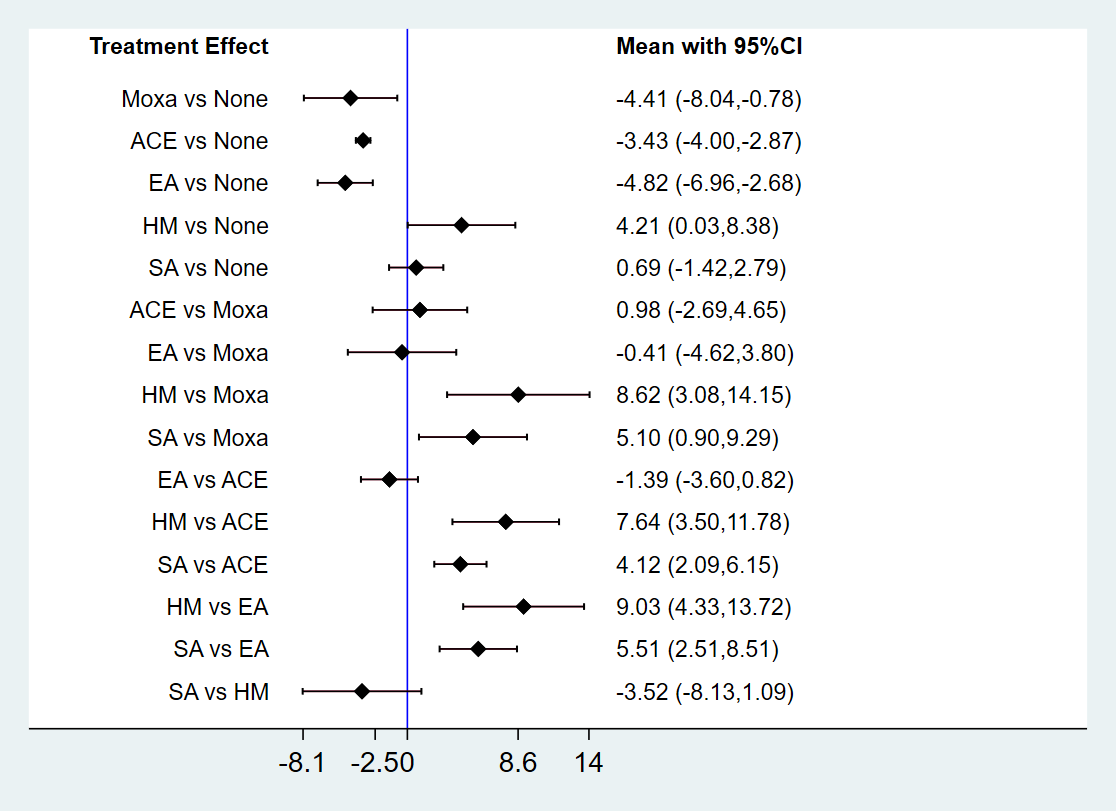


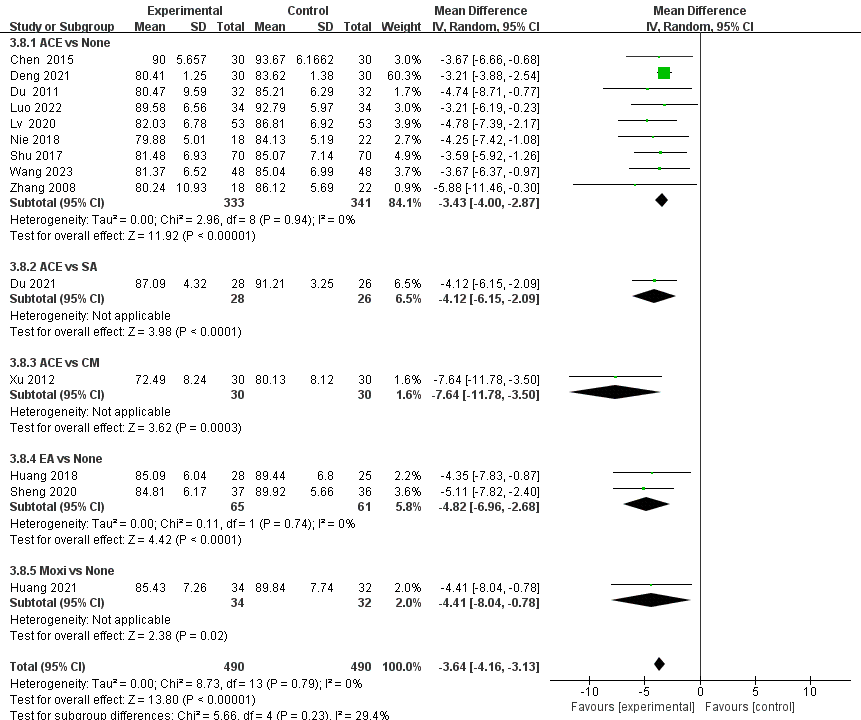


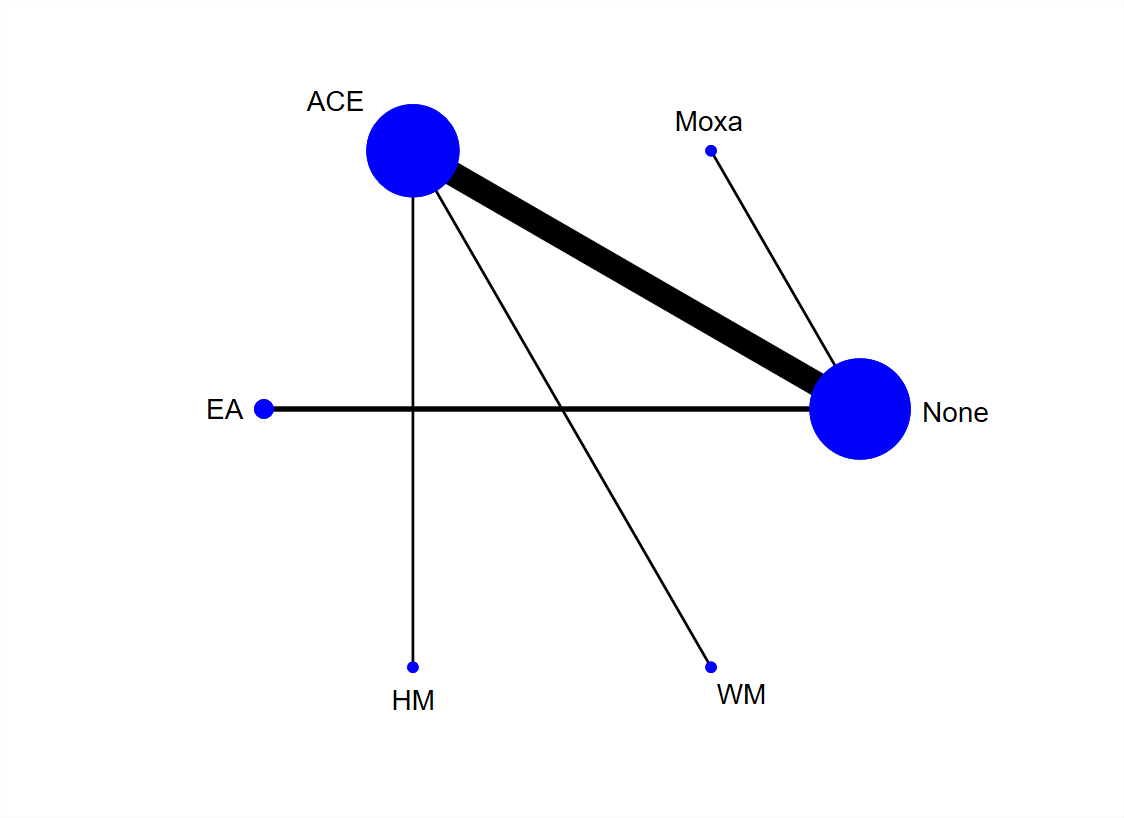


1. Body fat percentage


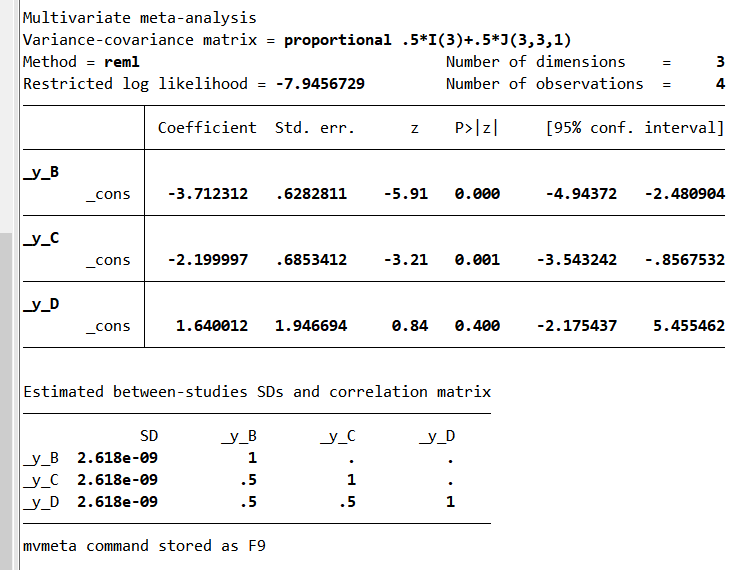


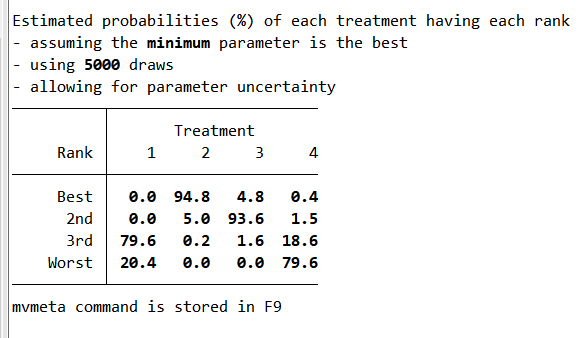


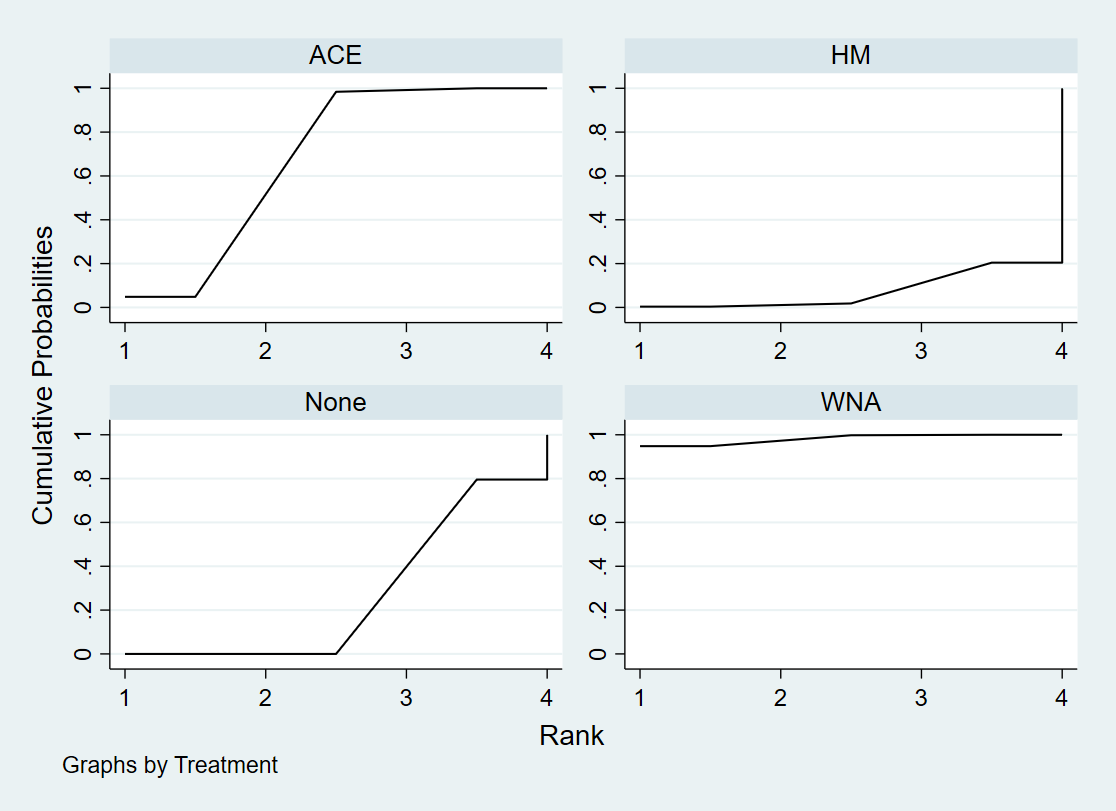


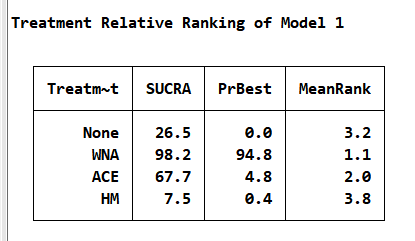


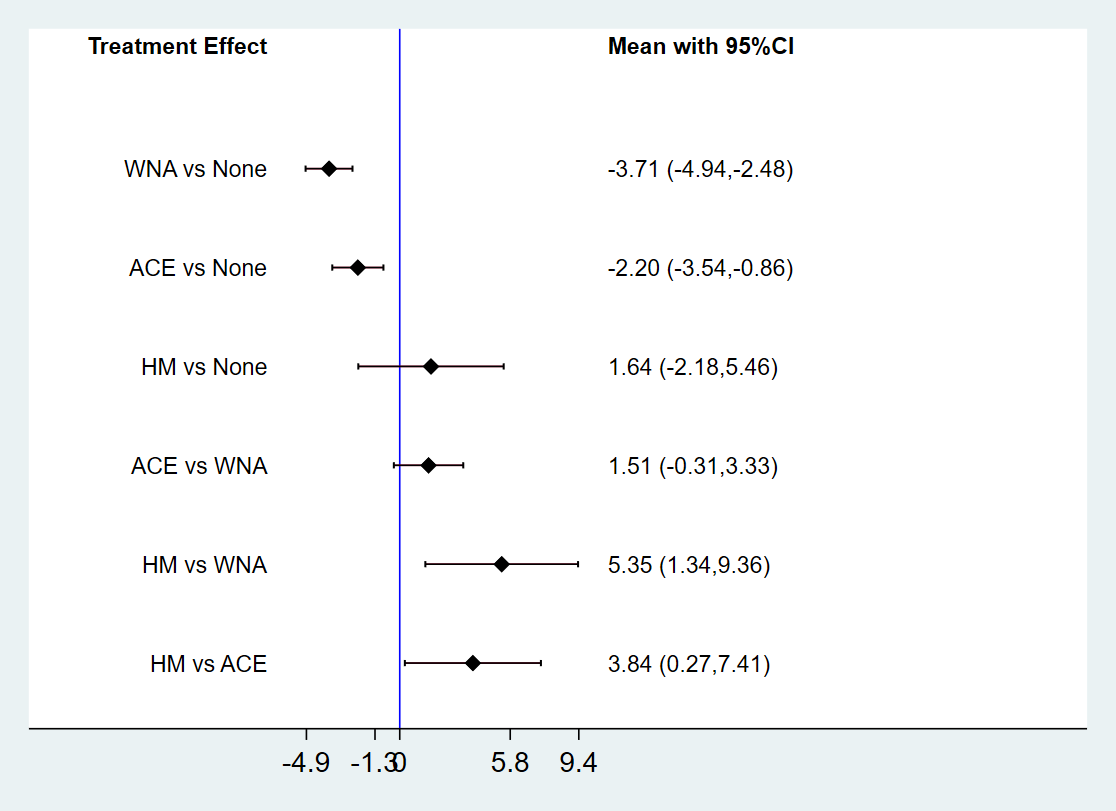

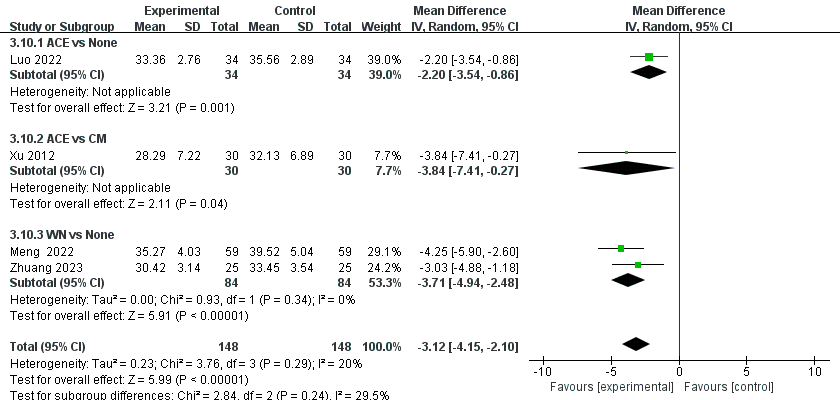


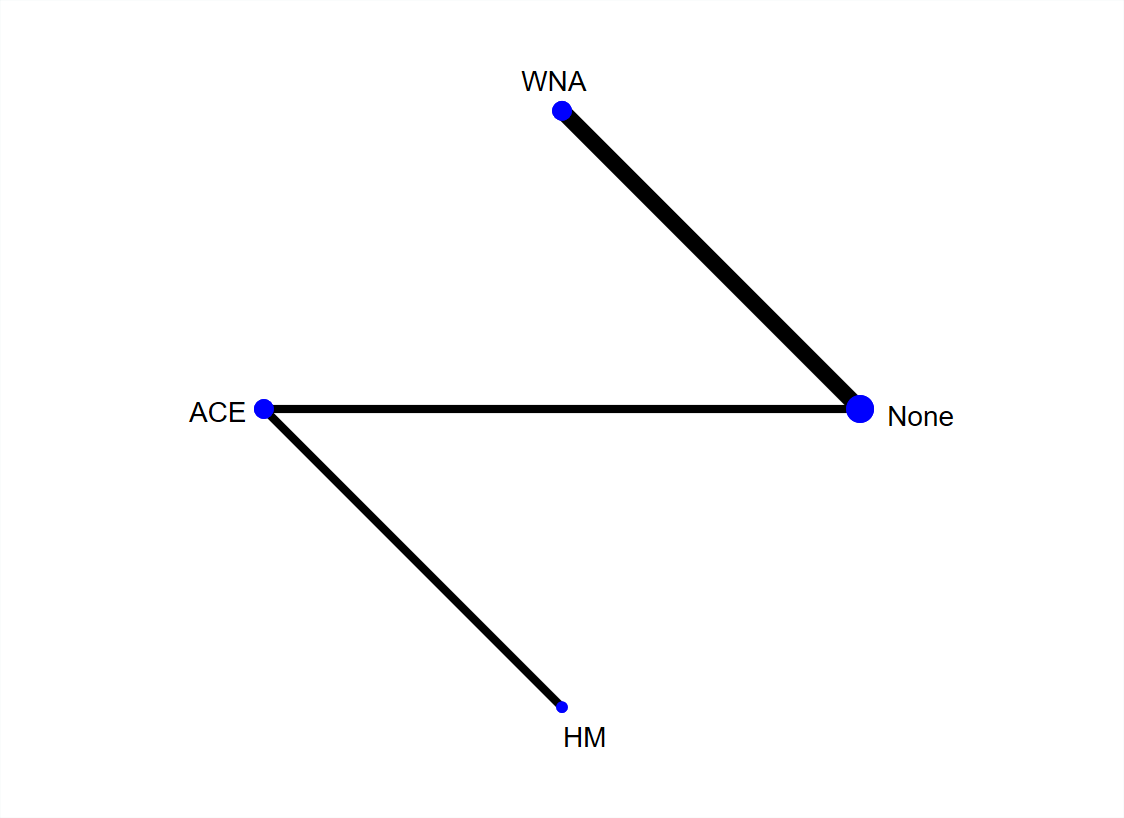


1. Kupperman


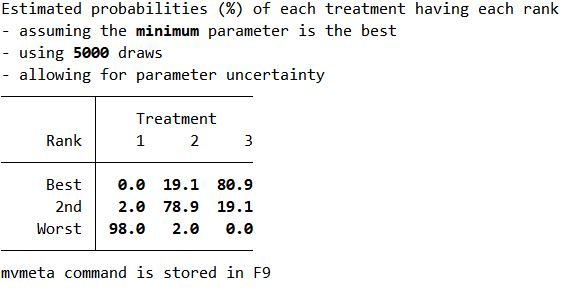


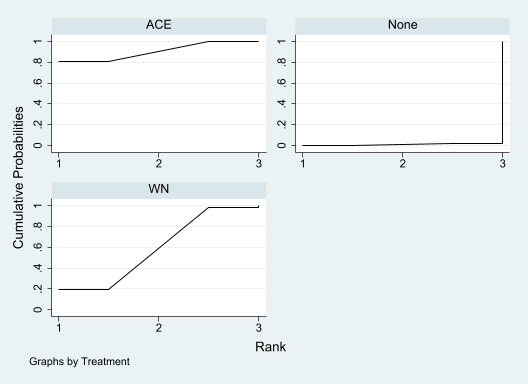


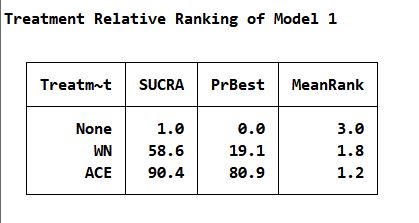


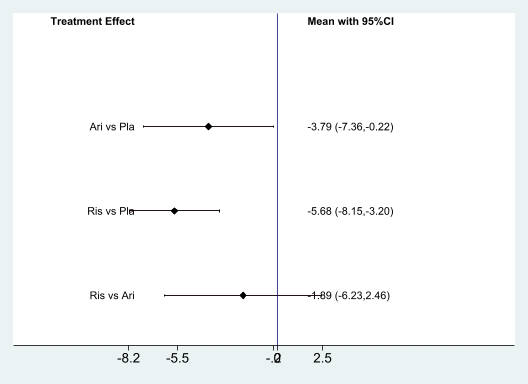


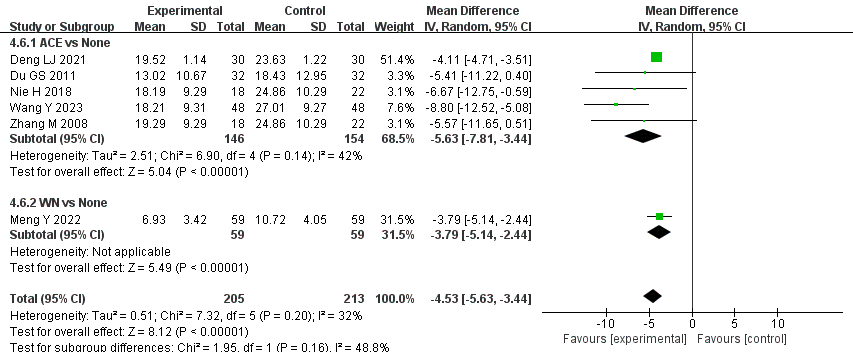


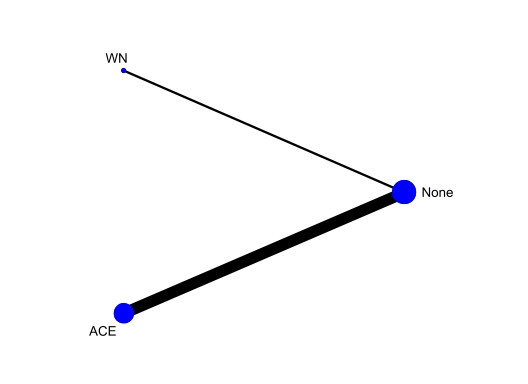


1. Other outcomes of interest

(1)E2


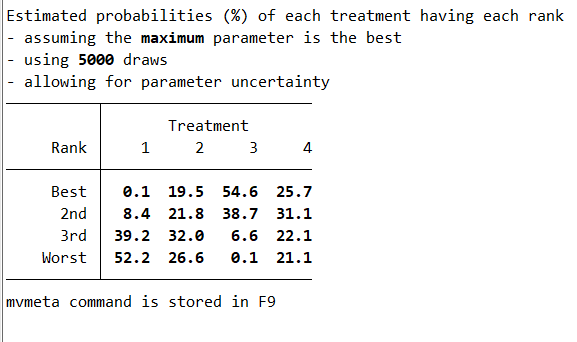


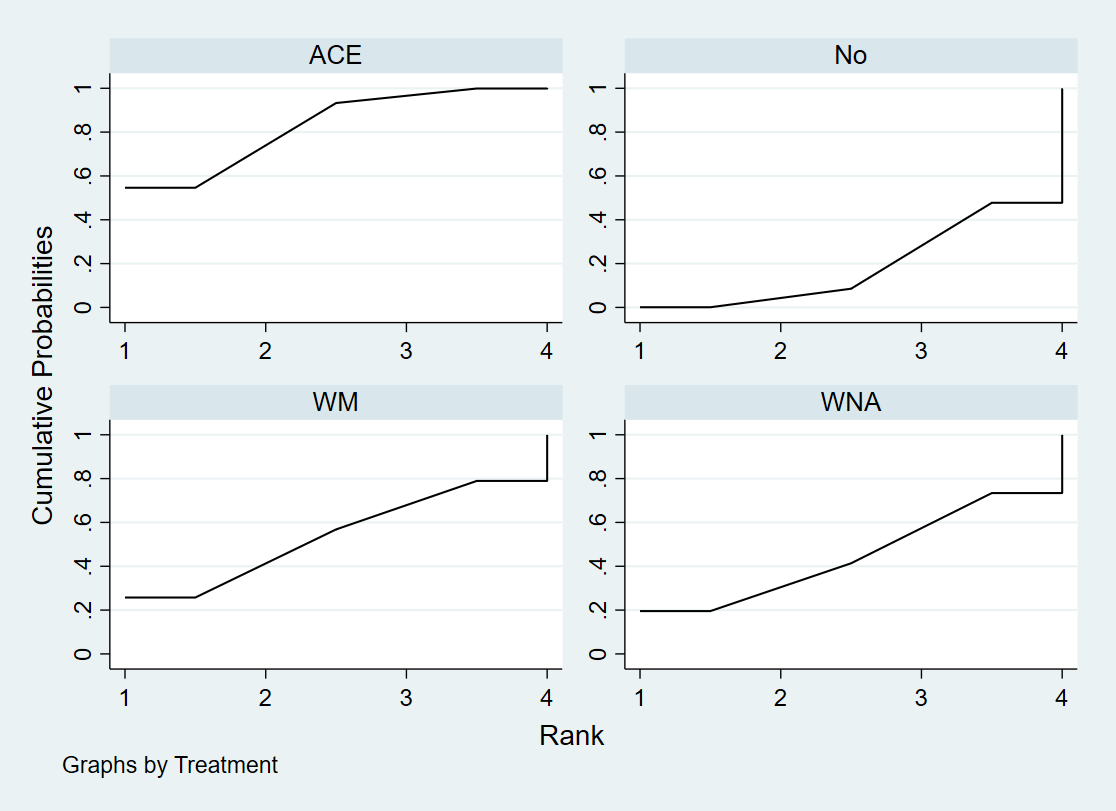


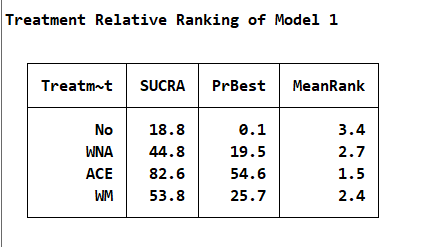


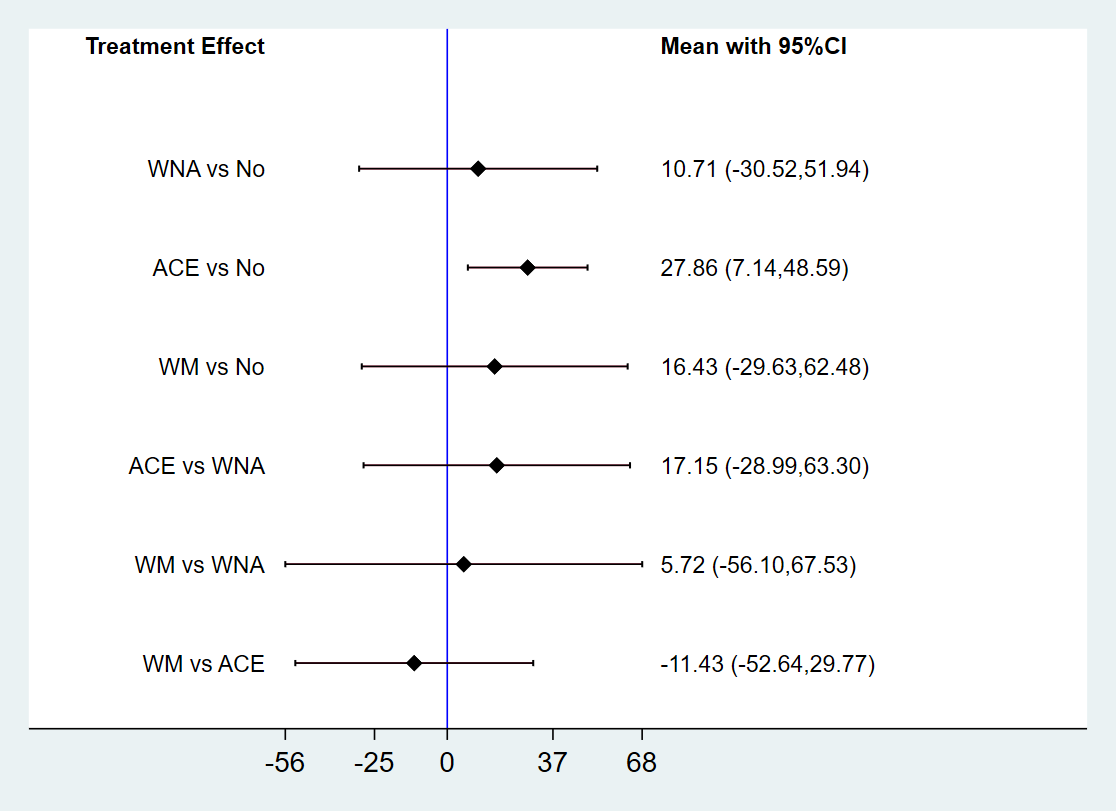

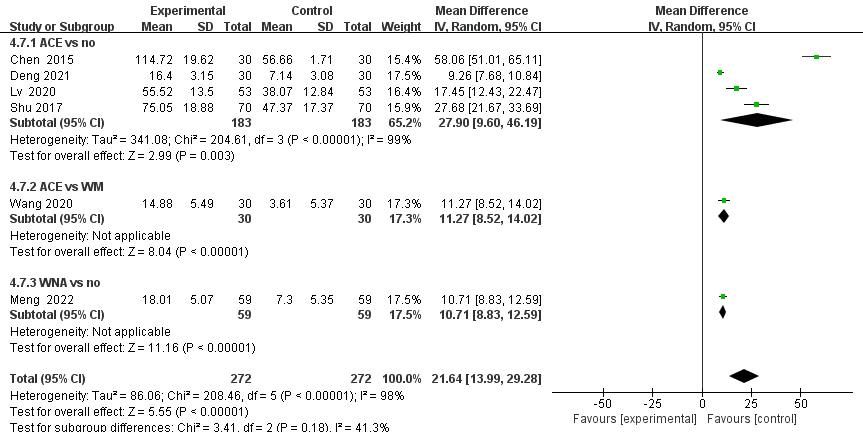


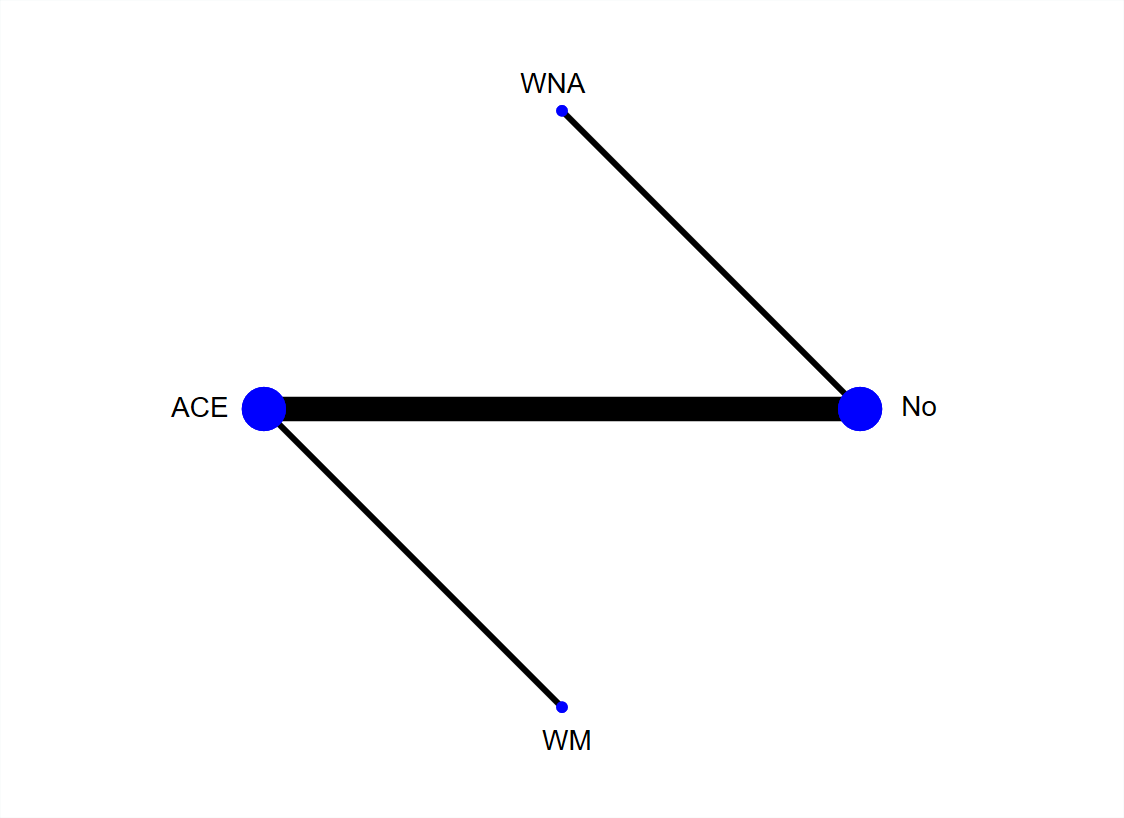


(2)FSH
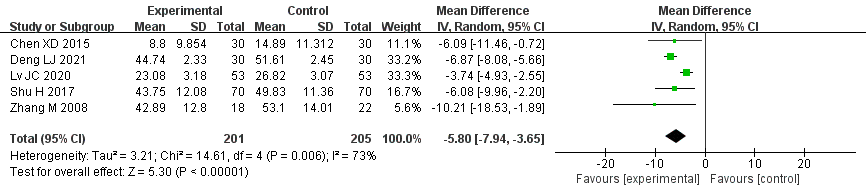


(3)LH
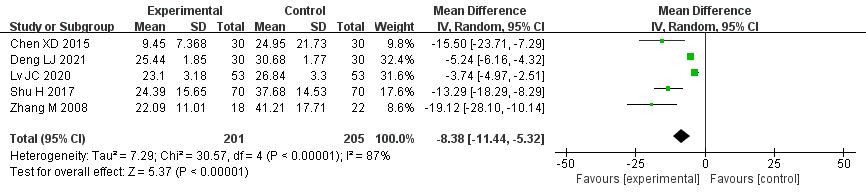

Supplement: Supplementary file 3 [file Supplementary_file_3.docx]
