# Supplementary material for: Comparison of efficacy of acupuncture-related therapy in the treatment of perimenopausal obesity: a network meta-analysis of randomized controlled trials
Source: Front Med (Lausanne). 2025 Nov 25;12:1642421. doi: 10.3389/fmed.2025.1642421 (PMC12685897; doi:10.3389/fmed.2025.1642421)
Supplement: Supplementary file 4 [file Supplementary_file_4.docx]

**Supplement S . Quality of evidence by the GRADE approach**

1. BMI

| Comparison | | Direct evidence  (pairwise meta-analysis) | Indirect evidence | Network meta- analysis |
| --- | --- | --- | --- | --- |
| ACE | None | Moderate  Risk of bias (- 1) | Moderate  Risk of bias (- 1) | Moderate  Risk of bias (- 1) |
| ACE | WN | - | Low  Risk of bias (- 1) Imprecision (- 1) | Low  Risk of bias (- 1) Imprecision (- 1) |
| ACE | LA | - | Low  Risk of bias (- 1) Imprecision (- 1) | Low  Risk of bias (- 1) Imprecision (- 1) |
| ACE | WM | Low  Risk of bias (- 1)  Imprecision (- 1) | Moderate  Risk of bias (- 1) | Moderate  Risk of bias (- 1) |
| ACE | EA | - | Low  Risk of bias (- 1) Imprecision (- 1) | Low  Risk of bias (- 1) Imprecision (- 1) |
| ACE | SA | Moderate  Risk of bias (- 1) | Moderate  Risk of bias (- 1) | Moderate  Risk of bias (- 1) |
| ACE | CM | Low  Risk of bias (- 1)  Imprecision (- 1) | Low  Risk of bias (- 1) Imprecision (- 1) | Low  Risk of bias (- 1) Imprecision (- 1) |
| EA | None | Low  Risk of bias (- 1)  Imprecision (- 1) | Low  Risk of bias (- 1) Imprecision (- 1) | Low  Risk of bias (- 1)  Imprecision (- 1) |
| EA | CM | - | Low  Risk of bias (- 1)  Imprecision (- 1) | Low  Risk of bias (- 1)  Imprecision (- 1) |
| EA | WM | Low  Risk of bias (- 1)  Imprecision (- 1) | Moderate  Risk of bias (- 1) | Moderate  Risk of bias (- 1) |
| WN | SA | - | Low  Risk of bias (- 1)  Imprecision (- 1) | Low  Risk of bias (- 1)  Imprecision (- 1) |
| WN | LA | - | Low  Risk of bias (- 1)  Imprecision (- 1) | Low  Risk of bias (- 1)  Imprecision (- 1) |
| WN | EA | - | Moderate  Risk of bias (- 1) | Moderate  Risk of bias (- 1) |
| WN | None | Moderate  Risk of bias (- 1) | Moderate  Risk of bias (- 1) | Moderate  Risk of bias (- 1) |
| WN | CM | - | Low  Risk of bias (- 1)  Imprecision (- 1) | Low  Risk of bias (- 1)  Imprecision (- 1) |
| WN | WM | - | Moderate  Risk of bias (- 1) | Moderate  Risk of bias (- 1) |
| LA | SA | - | Low  Risk of bias (- 1)  Imprecision (- 1) | Low  Risk of bias (- 1)  Imprecision (- 1) |
| LA | EA | - | Low  Risk of bias (- 1)  Imprecision (- 1) | Low  Risk of bias (- 1)  Imprecision (- 1) |
| LA | None | Moderate  Risk of bias (- 1) | Moderate  Risk of bias (- 1) | Moderate  Risk of bias (- 1) |
| LA | CM | - | Low  Risk of bias (- 1)  Imprecision (- 1) | Low  Risk of bias (- 1)  Imprecision (- 1) |
| LA | WM | - | Low  Risk of bias (- 1)  Imprecision (- 1) | Low  Risk of bias (- 1)  Imprecision (- 1) |
| SA | EA | - | Low  Risk of bias (- 1)  Imprecision (- 1) | Low  Risk of bias (- 1)  Imprecision (- 1) |
| SA | None | - | Moderate  Risk of bias (- 1) | Moderate  Risk of bias (- 1) |
| SA | CM | - | Low  Risk of bias (- 1)  Imprecision (- 1) | Low  Risk of bias (- 1)  Imprecision (- 1) |
| SA | WM | - | Low  Risk of bias (- 1)  Imprecision (- 1) | Low  Risk of bias (- 1)  Imprecision (- 1) |
| None | CM | - | Low  Risk of bias (- 1)  Imprecision (- 1) | Low  Risk of bias (- 1)  Imprecision (- 1) |
| None | WM | - | Low  Risk of bias (- 1)  Imprecision (- 1) | Low  Risk of bias (- 1)  Imprecision (- 1) |
| CM | WM | - | Low  Risk of bias (- 1)  Imprecision (- 1) | Low  Risk of bias (- 1)  Imprecision (- 1) |

(2) Body weight

| Comparison | | Direct evidence  (pairwise meta-analysis) | Indirect evidence | Network meta- analysis |
| --- | --- | --- | --- | --- |
| ACE | None | Low  Risk of bias (- 1)  Inconsistency (-1) | Moderate  Risk of bias (- 1) | Moderate  Risk of bias (- 1) |
| ACE | LA | - | Low  Risk of bias (- 1)  Imprecision (- 1) | Low  Risk of bias (- 1)  Imprecision (- 1) |
| ACE | TEAS | - | Low  Risk of bias (- 1)  Imprecision (- 1) | Low  Risk of bias (- 1)  Imprecision (- 1) |
| ACE | Moxibustion | - | Low  Risk of bias (- 1)  Imprecision (- 1) | Low  Risk of bias (- 1)  Imprecision (- 1) |
| ACE | CM | Moderate  Risk of bias (- 1) | Low  Risk of bias (- 1)  Imprecision (- 1) | Moderate  Risk of bias (- 1) |
| ACE | SA | Low  Risk of bias (- 1)  Imprecision (- 1) | Low  Risk of bias (- 1)  Imprecision (- 1) | Low  Risk of bias (- 1)  Imprecision (- 1) |
| ACE | EA | - | Low  Risk of bias (- 1)  Imprecision (- 1) | Low  Risk of bias (- 1)  Imprecision (- 1) |
| ACE | WM | - | Low  Risk of bias (- 1)  Imprecision (- 1) | Low  Risk of bias (- 1)  Imprecision (- 1) |
| LA | TEAS | - | Low  Risk of bias (- 1)  Imprecision (- 1) | Low  Risk of bias (- 1)  Imprecision (- 1) |
| LA | Moxibustion | - | Low  Risk of bias (- 1)  Imprecision (- 1) | Low  Risk of bias (- 1)  Imprecision (- 1) |
| LA | SA | - | Low  Risk of bias (- 1)  Imprecision (- 1) | Low  Risk of bias (- 1)  Imprecision (- 1) |
| LA | EA | - | Low  Risk of bias (- 1)  Imprecision (- 1) | Low  Risk of bias (- 1)  Imprecision (- 1) |
| LA | None | Moderate  Risk of bias (- 1) | Low  Risk of bias (- 1)  Imprecision (- 1) | Moderate  Risk of bias (- 1) |
| LA | CM | - | Low  Risk of bias (- 1)  Imprecision (- 1) | Low  Risk of bias (- 1)  Imprecision (- 1) |
| LA | WM | - | Low  Risk of bias (- 1)  Imprecision (- 1) | Low  Risk of bias (- 1)  Imprecision (- 1) |
| EA | None | Low  Risk of bias (- 1)  Imprecision (- 1) | Low  Risk of bias (- 1)  Imprecision (- 1) | Low  Risk of bias (- 1)  Imprecision (- 1) |
| EA | WM | Low  Risk of bias (- 1)  Imprecision (- 1) | Low  Risk of bias (- 1)  Imprecision (- 1) | Low  Risk of bias (- 1)  Imprecision (- 1) |
| EA | CM | - | Low  Risk of bias (- 1)  Imprecision (- 1) | Low  Risk of bias (- 1)  Imprecision (- 1) |
| Moxibustion | None | Low  Risk of bias (- 1)  Imprecision (- 1) | Low  Risk of bias (- 1)  Imprecision (- 1) | Low  Risk of bias (- 1)  Imprecision (- 1) |
| Moxibustion | SA | - | Low  Risk of bias (- 1)  Imprecision (- 1) | Low  Risk of bias (- 1)  Imprecision (- 1) |
| Moxibustion | EA | - | Low  Risk of bias (- 1)  Imprecision (- 1) | Low  Risk of bias (- 1)  Imprecision (- 1) |
| Moxibustion | CM | - | Low  Risk of bias (- 1)  Imprecision (- 1) | Low  Risk of bias (- 1)  Imprecision (- 1) |
| Moxibustion | WM | - | Low  Risk of bias (- 1)  Imprecision (- 1) | Low  Risk of bias (- 1)  Imprecision (- 1) |
| TEAS | Moxibustion | - | Low  Risk of bias (- 1)  Imprecision (- 1) | Low  Risk of bias (- 1)  Imprecision (- 1) |
| TEAS | SA | - | Low  Risk of bias (- 1)  Imprecision (- 1) | Low  Risk of bias (- 1)  Imprecision (- 1) |
| TEAS | EA | - | Low  Risk of bias (- 1)  Imprecision (- 1) | Low  Risk of bias (- 1)  Imprecision (- 1) |
| TEAS | None | Low  Risk of bias (- 1)  Imprecision (- 1) | Low  Risk of bias (- 1)  Imprecision (- 1) | Low  Risk of bias (- 1)  Imprecision (- 1) |
| TEAS | CM | - | Low  Risk of bias (- 1)  Imprecision (- 1) | Low  Risk of bias (- 1)  Imprecision (- 1) |
| TEAS | WM | - | Low  Risk of bias (- 1)  Imprecision (- 1) | Low  Risk of bias (- 1)  Imprecision (- 1) |
| SA | EA | - | Low  Risk of bias (- 1)  Imprecision (- 1) | Low  Risk of bias (- 1)  Imprecision (- 1) |
| SA | None | - | Low  Risk of bias (- 1)  Imprecision (- 1) | Low  Risk of bias (- 1)  Imprecision (- 1) |
| SA | CM | - | Low  Risk of bias (- 1)  Imprecision (- 1) | Low  Risk of bias (- 1)  Imprecision (- 1) |
| SA | WM | - | Low  Risk of bias (- 1)  Imprecision (- 1) | Low  Risk of bias (- 1)  Imprecision (- 1) |
| None | CM | - | Low  Risk of bias (- 1)  Imprecision (- 1) | Low  Risk of bias (- 1)  Imprecision (- 1) |
| None | WM | - | Low  Risk of bias (- 1)  Imprecision (- 1) | Low  Risk of bias (- 1)  Imprecision (- 1) |
| CM | WM | - | Low  Risk of bias (- 1)  Imprecision (- 1) | Low  Risk of bias (- 1)  Imprecision (- 1) |

（3）waist circumference

| Comparison | | Direct evidence  (pairwise meta-analysis) | Indirect evidence | Network meta- analysis |
| --- | --- | --- | --- | --- |
| LA | EA | - | Low  Risk of bias (- 1)  Imprecision (- 1) | Low  Risk of bias (- 1)  Imprecision (- 1) |
| LA | Moxibustion | - | Low  Risk of bias (- 1)  Imprecision (- 1) | Low  Risk of bias (- 1)  Imprecision (- 1) |
| LA | ACE | - | Low  Risk of bias (- 1)  Imprecision (- 1) | Low  Risk of bias (- 1)  Imprecision (- 1) |
| LA | None | Moderate  Risk of bias (- 1) | Low  Risk of bias (- 1)  Imprecision (- 1) | Moderate  Risk of bias (- 1) |
| LA | TEAS | - | Low  Risk of bias (- 1)  Imprecision (- 1) | Low  Risk of bias (- 1)  Imprecision (- 1) |
| LA | SA | - | Low  Risk of bias (- 1)  Imprecision (- 1) | Low  Risk of bias (- 1)  Imprecision (- 1) |
| LA | CM | - | Low  Risk of bias (- 1)  Imprecision (- 1) | Low  Risk of bias (- 1)  Imprecision (- 1) |
| ACE | None | Moderate  Risk of bias (- 1) | Moderate  Risk of bias (- 1) | Moderate  Risk of bias (- 1) |
| ACE | TEAS | - | Low  Risk of bias (- 1)  Imprecision (- 1) | Low  Risk of bias (- 1)  Imprecision (- 1) |
| ACE | SA | Moderate  Risk of bias (- 1) | Moderate  Risk of bias (- 1) | Moderate  Risk of bias (- 1) |
| ACE | CM | Low  Risk of bias (- 1)  Imprecision (- 1) | Low  Risk of bias (- 1)  Imprecision (- 1) | Low  Risk of bias (- 1)  Imprecision (- 1) |
| EA | Moxibustion | - | Low  Risk of bias (- 1)  Imprecision (- 1) | Low  Risk of bias (- 1)  Imprecision (- 1) |
| EA | ACE | - | Low  Risk of bias (- 1)  Imprecision (- 1) | Low  Risk of bias (- 1)  Imprecision (- 1) |
| EA | TEAS | - | Low  Risk of bias (- 1)  Imprecision (- 1) | Low  Risk of bias (- 1)  Imprecision (- 1) |
| EA | None | Moderate  Risk of bias (- 1) | Moderate  Risk of bias (- 1) | Moderate  Risk of bias (- 1) |
| EA | SA | - | Low  Risk of bias (- 1)  Imprecision (- 1) | Low  Risk of bias (- 1)  Imprecision (- 1) |
| EA | CM | - | Low  Risk of bias (- 1)  Imprecision (- 1) | Low  Risk of bias (- 1)  Imprecision (- 1) |
| Moxibustion | ACE | - | Low  Risk of bias (- 1)  Imprecision (- 1) | Low  Risk of bias (- 1)  Imprecision (- 1) |
| Moxibustion | TEAS | - | Low  Risk of bias (- 1)  Imprecision (- 1) | Low  Risk of bias (- 1)  Imprecision (- 1) |
| Moxibustion | None | Low  Risk of bias (- 1)  Imprecision (- 1) | Low  Risk of bias (- 1)  Imprecision (- 1) | Low  Risk of bias (- 1)  Imprecision (- 1) |
| Moxibustion | SA | - | Low  Risk of bias (- 1)  Imprecision (- 1) | Low  Risk of bias (- 1)  Imprecision (- 1) |
| Moxibustion | CM | - | Low  Risk of bias (- 1)  Imprecision (- 1) | Low  Risk of bias (- 1)  Imprecision (- 1) |
| TEAS | None | Low  Risk of bias (- 1)  Imprecision (- 1) | Low  Risk of bias (- 1)  Imprecision (- 1) | Low  Risk of bias (- 1)  Imprecision (- 1) |
| TEAS | SA | - | Low  Risk of bias (- 1)  Imprecision (- 1) | Low  Risk of bias (- 1)  Imprecision (- 1) |
| TEAS | CM | - | Low  Risk of bias (- 1)  Imprecision (- 1) | Low  Risk of bias (- 1)  Imprecision (- 1) |
| SA | CM | - | Low  Risk of bias (- 1)  Imprecision (- 1) | Low  Risk of bias (- 1)  Imprecision (- 1) |
| None | SA | - | Low  Risk of bias (- 1)  Imprecision (- 1) | Low  Risk of bias (- 1)  Imprecision (- 1) |
| None | CM | - | Low  Risk of bias (- 1)  Imprecision (- 1) | Low  Risk of bias (- 1)  Imprecision (- 1) |

(4)Body fat rate

| Comparison | | Direct evidence  (pairwise meta-analysis) | Indirect evidence | Network meta- analysis |
| --- | --- | --- | --- | --- |
| TEAS | WM | - | Low  Risk of bias (- 1)  Imprecision (- 1) | Low  Risk of bias (- 1)  Imprecision (- 1) |
| TEAS | ACE | - | Low  Risk of bias (- 1)  Imprecision (- 1) | Low  Risk of bias (- 1)  Imprecision (- 1) |
| TEAS | None | Moderate  Risk of bias (- 1) | Moderate  Risk of bias (- 1) | Moderate  Risk of bias (- 1) |
| TEAS | CM | - | Low  Risk of bias (- 1)  Imprecision (- 1) | Low  Risk of bias (- 1)  Imprecision (- 1) |
| WN | ACE | - | Low  Risk of bias (- 1)  Imprecision (- 1) | Low  Risk of bias (- 1)  Imprecision (- 1) |
| WN | None | Moderate  Risk of bias (- 1) | Moderate  Risk of bias (- 1) | Moderate  Risk of bias (- 1) |
| WN | CM | - | Low  Risk of bias (- 1)  Imprecision (- 1) | Low  Risk of bias (- 1)  Imprecision (- 1) |
| ACE | None | Low  Risk of bias (- 1)  Imprecision (- 1) | Low  Risk of bias (- 1)  Imprecision (- 1) | Low  Risk of bias (- 1)  Imprecision (- 1) |
| ACE | CM | Low  Risk of bias (- 1)  Imprecision (- 1) | Low  Risk of bias (- 1)  Imprecision (- 1) | Low  Risk of bias (- 1)  Imprecision (- 1) |
| None | CM | - | Low  Risk of bias (- 1)  Imprecision (- 1) | Low  Risk of bias (- 1)  Imprecision (- 1) |

（5）Kupperman

| Comparison | | Direct evidence  (pairwise meta-analysis) | Indirect evidence | Network meta- analysis |
| --- | --- | --- | --- | --- |
| ACE | WN | Moderate  Risk of bias (- 1) |  | Moderate  Risk of bias (- 1) |
| ACE | None | - |  | Low  Risk of bias (- 1)  Imprecision (- 1) |
| WN | None | Moderate  Risk of bias (- 1) |  | Moderate  Risk of bias (- 1) |
